# Ramón y Cajal’s ‘lenticular tract’ represents infrasubthalamic pyramidal collaterals targeting mesodiencephalic centers: an obscure misunderstood aspect of the motor pathway clarified by Allen Mouse Brain Connectivity data

**DOI:** 10.3389/fnana.2024.1500181

**Published:** 2024-11-18

**Authors:** Luis Puelles

**Affiliations:** Department of Human Anatomy, School of Medicine, University of Murcia, and Pascual Parrilla Murcia Institute of Biomedical Research, Murcia, Spain

**Keywords:** forebrain tracts, pyramidal collaterals, motor pathway, preoculomotor nuclei, zona incerta, posterior intralaminar nucleus, superior colliculus, BSC nucleus

## Abstract

Ramón y Cajal repeatedly described a diencephalic tegmental tract in mammals, which he wrongly identified as corresponding to Forel’s lenticular tract, while insisting it was formed by fasciculated infrasubthalamic collaterals of the pyramidal tract. These fibers circulated backwards between the zona incerta and the substantia nigra and were lost in the rubral neighborhood, so that their targets remained unknown. The modern literature does not register these pyramidal collaterals at all. In this report, anterograde axon-tracing experiments with motor cortex EGFP injections available at the Allen Mouse Brain Connectivity database were studied to assess whether Ramón y Cajal’s collateral tract exists in the mouse and, in that case, examine the issue of its missing targets. All Allen motor cortex experiments showed straightforwardly the tract described by Ramón y Cajal, which he had lost at midcourse because it diverges into the mesodiencephalic alar plate. Moreover, it was observed to bifurcate as it enters the diencephalon into a deep medial component not distinguished by Ramón y Cajal, which targets diencephalic and mesencephalic preoculomotor cell populations and the deep ZI and Forel’s field, and a thicker outer component, the part he described, which ascends through intermediate thalamic and pretectal alar structures (PIL, RETh, JcRt, and CoRt) until it ends densely on the paratectal bed nucleus of the brachium of the superior colliculus (BSC). The latter is frequently misinterpreted as a lateral part of the SC. Other midbrain targets included the rubral area, the MRt, a novel bilateral reticular endorubral field (ERF), and the preisthmic cuneiform nucleus (Cnf).

## Introduction

Initial description of the forebrain *lenticular fasciculus* by Forel (1877, 1907) led to further use of this concept by Déjerine (1895), Von Köllicker (1902), von Monakow (1895), Baker (1909), Foix and Nicolesco (1925), and Winkler (1933). These diverse descriptions were based mainly on carmine and myelin preparations and were by no means in total mutual agreement or accurate as regards modern notions obtained experimentally. In particular, Ramón y Cajal (1900, 1903, 1911) produced a discrepant account of the origin and initial course of what he wrongly thought was Forel’s lenticular tract on the basis of Golgi and Ehrlich (methylene blue) staining data. He repeatedly identified as ‘Forel’s lenticular tract’, a compact packet of fibers coursing through the prerubral tegmentum which he thought ‘probably originates as collaterals of the motor cortex efferent pathway’ (Ramón y Cajal, 1900, 1903, 1911, 1928, 1966). He lost track of these fibers in the neighborhood of the nucleus ruber. Ulterior neuroanatomic findings revealed that these fibers *could not be* Forel’s lenticular fasciculus (which is definitely a pallido-thalamic connection; Forel himself had thought it was a rubro-striatal connection). In any case the terminal target of Ramón y Cajal’s pyramidal collaterals remained unexplored, nor was their existence as true pyramidal collaterals corroborated by other scientists. In the present report, I re-examine this forgotten historic issue using material from the Allen Mouse Brain Connectivity Atlas.^1^ These data abundantly corroborate Ramón y Cajal’s description and, importantly, identify its apparent targets.

### Historic background

Forel (1877) first described his ‘tegmental fields H1 and H2’ (the H stands for Haube = tegmentum in German), referring to myelinated tracts that crisscross the diencephalic prerubral tegmentum. One of these tracts was the *lenticular fasciculus*, which perforates the peduncle and was interpreted tentatively by Forel as a rubro-striatal connection. The descriptor ‘lenticular tract’ owed indeed to Forel’s wrong belief that this tract extends from the red nucleus across the peduncle into the striato-pallidal lenticular nucleus complex. Its inverse origin in the ‘corpus striatum’ (which term initially encompassed both striatal and pallidal components), or strictly, within the internal part of the globus pallidum (see Puelles et al., 2023 in regard to its genoarchitectonic nature in rodents), was ascertained subsequently. This interpretation was indeed postulated by von Monakow (1895) and others—see reviews in Foix and Nicolesco (1925) and Nauta and Mehler (1966). Déjerine (1895) distinctly mapped this tract in the human as myelinated fibers separating the subthalamic nucleus from the zona incerta (his Figures 42, 45, 325, and 329 in Vol. 2), although he supported Forel’s wrong interpretation about their rubral origin.

**Figure 1:**
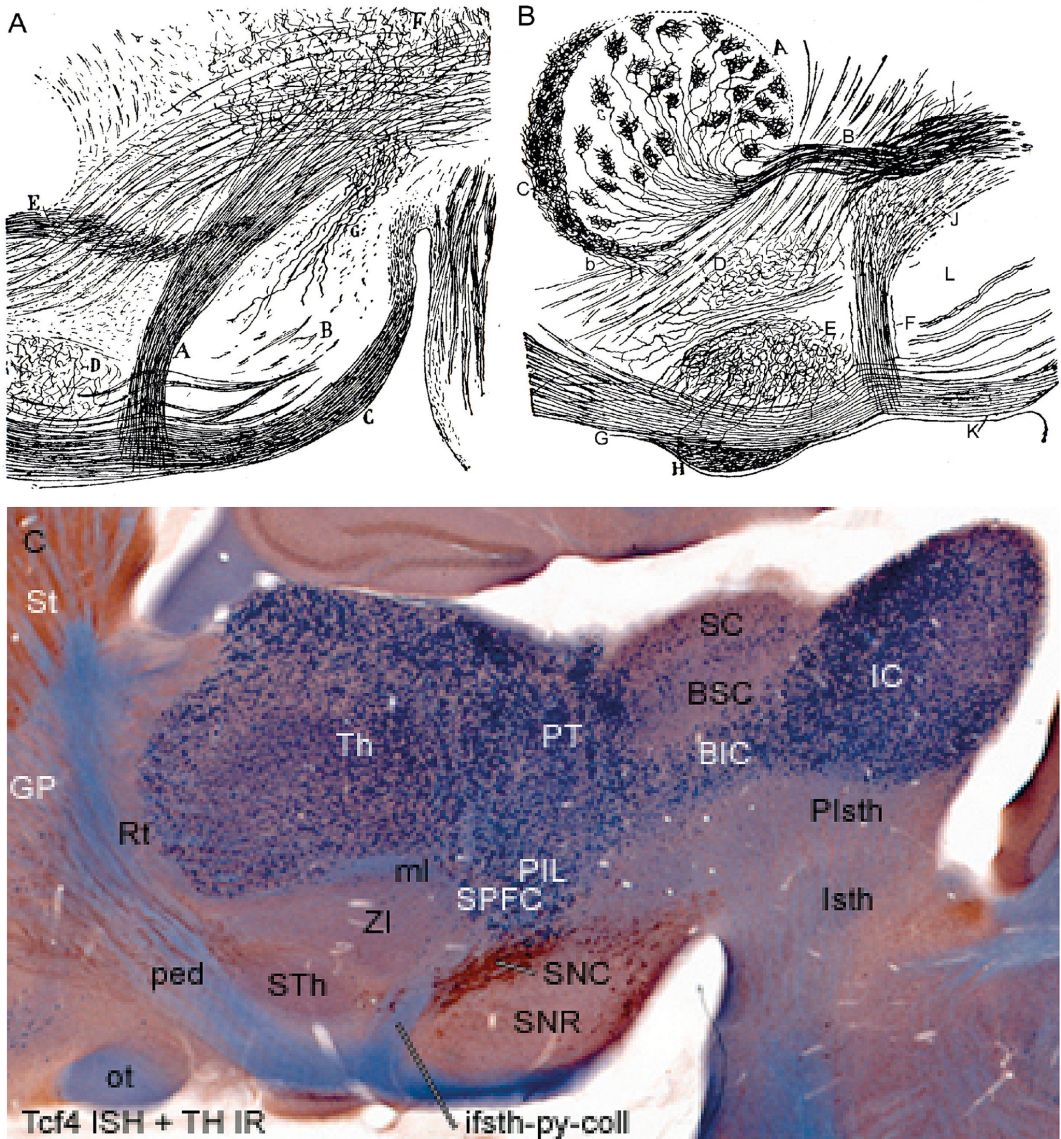
Histologic illustration of the ifsth-py-coll tract in Ramón y Cajal’s material, compared with corroborating recent genoarchitectonic data. **(A,B)** Reproduction with slight modification of Figures 194 and 21, respectively, of Ramón y Cajal (1966), illustrating his Golgi-stained infrasubthalamic pyramidal collateral tract in sagittal sections. **(A)** Corresponds to an adult mouse, whereas **(B)** is from a P20 specimen, **(C)** is a similar sagittal section (100 micrometer-thick) of an adult mouse from our collection that was reacted for *Tcf4 in situ* hybridization reaction and counterstained with tyrosine hydroxylase (TH) immunoreaction. In the three images **(A–C)** rostral is oriented to the left and dorsal is up. **(A)** shows a detail view of the ifsth-py-coll tract (A) as it originates from the peduncle (tagged C) between the subthalamic nucleus (D) and the substantia nigra (B). Its fibers apparently enter Forel’s field under the thalamus. The medial lemniscus entering the thalamus is also represented (E). The zona incerta was not identified (but see **B**). **(B)** is another sagittal section showing the hypothalamic course of the peduncle (G) bending into its diencephalic tegmental continuation (K). Immediately under the subthalamic nucleus (E), the ifsth-py-coll tract originates (F) and crosses partially the substantia nigra (L) to enter the interstice that separates the latter from the zona incerta (D). The fibers approach caudalwards the medial lemniscus (B) as the latter enters the thalamus (A,C). The author identifies the area labeled (J) as the field of Forel. (H) is identified in the original caption as the optic tract, but it probably corresponds instead to the medial end of the temporal hippocampus [compare with the distinct optic tract—ot—in **(C)**]. The adult mouse parasagittal section labeled with *Tcf4* and TH in (C) illustrates the same area of the brain as well as the lateral part of the midbrain, relevantly for our present study. *Tcf4* ISH labels all thalamic and pretectal alar neurons (i.e., massive blue labeling of Th and PT) and some midbrain ones. The bed nucleus of the brachium of the inferior colliculus—BIC—and the inferior colliculus—IC—are strongly labeled, whereas the superior colliculus—SC—is sparsely labeled; the paratectal bed nucleus of the brachium of the superior colliculus (BSC) appears largely unlabeled (compare with Puelles (2022); Figure 1 therein). The ISH reaction also produces some unspecific background reaction in myelinated tracts (ped. ot, ml). TH-IR labels the substantia nigra compacta neurons (SNC), some nigrostriatal fibers, and the striatum neuropile (St). Note the ifsth-py-coll tract arises from the peduncle (ped) between the subthalamic nucleus (STh) and the substantia nigra (SNC, SNR), and its relationship with the neighboring zona incerta (ZI) and medial lemniscus (ml) coincides with the Ramón y Cajal drawings A and B. The subparafascicular and posterior intralaminar thalamic nuclei (SPFC, PIL) were not identified in those drawings. Note the real pallido-thalamic lenticular tract crosses the peduncle above the STh, roughly where the tag ‘ped’ appears in panel **(C)**.

On several occasions, Ramón y Cajal (1900, 1903, 1911, 1928, 1966) reported observations on Forel’s lenticular tract in the mouse, rat, rabbit, guinea pig, cat, dog, and man. Contrarily to Forel and Déjerine, he always interpreted the tegmental fasciculated fibers he observed as corresponding to collaterals of the motor pyramidal tract (i.e., the middle part of the cerebral peduncle) emitted caudalwards at right angles just underneath the subthalamic nucleus. He illustrated the peduncular origin of such collaterals with both Golgi and Ehrlich methods (e.g., Ramón y Cajal, 1911, 1966; text starting p. 185; see his Figures 21, 24, 29, 66, 67, 100, 101, 102, 103, 104, and 111). According to Ramón y Cajal, these collaterals pass caudalwards under the zona incerta into the ‘diencephalic tegmentum’ (note the zona incerta is presently understood as a prethalamic alar formation; Puelles et al., 2012a, 2012b, 2021). In doing so, the fibers first pass through the gap existing between the subthalamic nucleus and the prethalamic rostral pole of the substantia nigra (Figures 1A–C). The fibers clearly reach Forel’s H2 field and the lateral rubral neighborhood (rubral capsule) in p1, where they were lost.

Experimental data first obtained with the Marchi method confirmed subsequently that basal ganglia output arising from the internal division of the globus pallidus consists of two different pathways, namely, the lenticular fasciculus of Forel and the ansa lenticularis (the former perforates the peduncle, whereas the ansa curves around it). Both tracts converge at the level of Forel’s field H1, giving rise to the thalamic fasciculus (Forel’s field H2). Forel’s lenticular tract is accordingly understood as a transpeduncular (perforant) tract mediating internal globus pallidus input to the motor thalamus. This notion was further corroborated using Nauta and Gygax degeneration method variants [see historic reviews in Nauta and Mehler (1966) and Nauta (1993)]. It is under this later concept how the *lenticular fascicle or tract* is presently described within basal ganglia circuitry in all current neuroanatomical textbooks or treatises (e.g., Crosby et al., 1962; Brodal, 1981; Nieuwenhuys et al., 2008; Parent and Parent, 2016).

It seems clear in retrospect that Ramón y Cajal misinterpreted *a different tract* arising out of the final infrasubthalamic hypothalamic course of the peduncle as the lenticular fascicle of Forel, whose transpeduncular course is rather suprasubthalamic (Ramón y Cajal, 1900, 1903, 1911, 1928, 1966). He mainly studied this tract in small mammals, in which Forel’s fields are less distinct histologically than in primates and man. He drew and described repeatedly a tract that he credibly interpreted as a packet of collaterals that sprouts caudalwards out of the pyramidal tract just under the subthalamic nucleus, near the substantia nigra (e.g., Ramón y Cajal, 1966; see his text starting at p. 185 and his Figures 104 and 21, reproduced in our Figures 1A,B). To evade confusion with the real lenticular tract, I will henceforth refer to Ramón y Cajal’s tract as ‘*infrasubthalamic pyramidal collateral tract*’ (ifsth-py-coll; Figures 1A–C).

**Figure 2:**
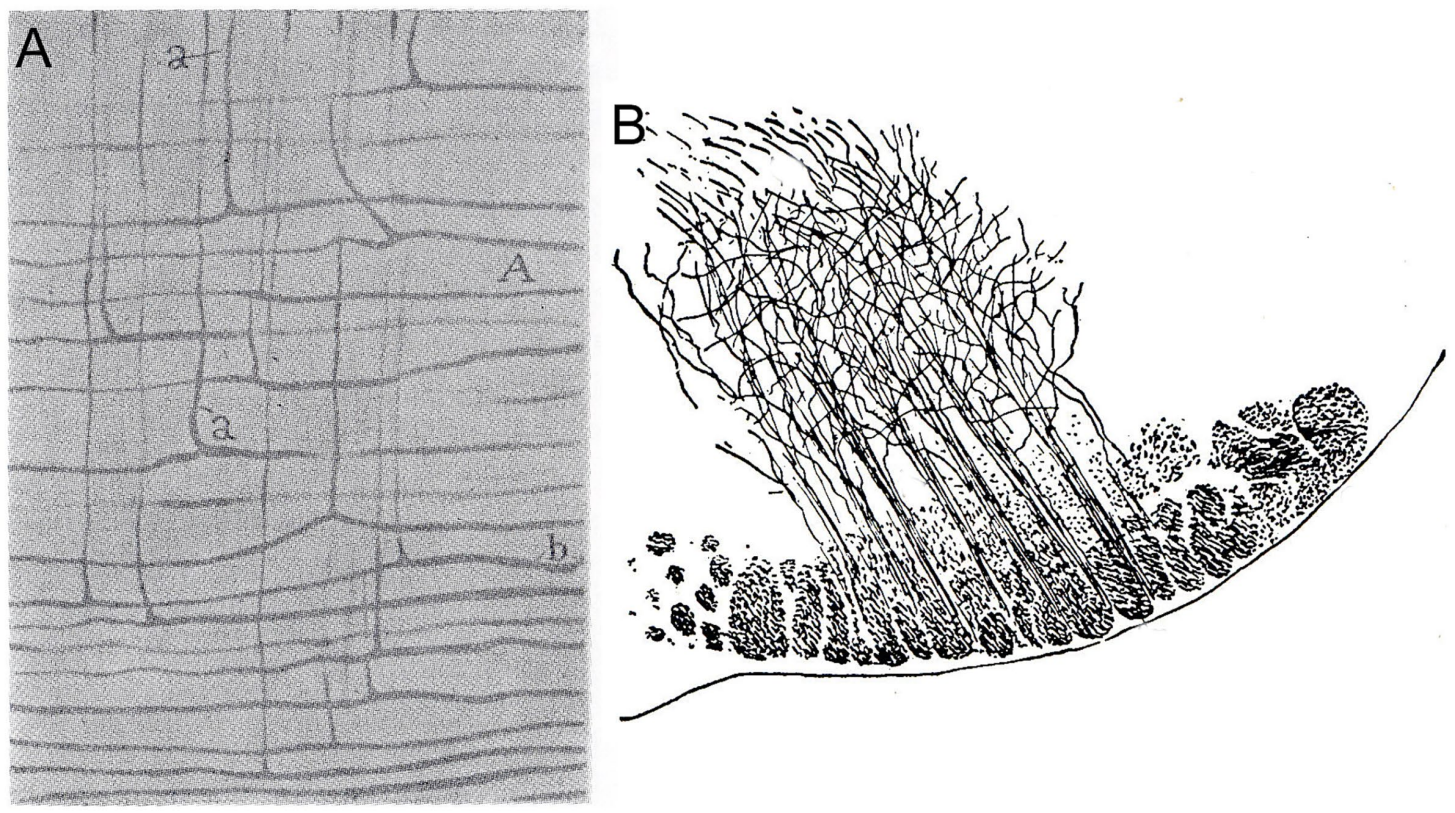
Detail drawings of the ifsth-py-coll tract origin from Ramón y Cajal’s (1966) Figures 103 and 101, corresponding, respectively, to methylene blue (Ehrlich method) impregnation in the cat **(A)** and Golgi impregnation in a P15 mouse **(B)**. **(A)** Shows peduncular fibers (tagged A) coursing from left to right and infrasubthalamic collaterals (tagged ‘a’) arising perpendicularly and coursing uniformly upwards. **(B)** Shows a cross section of the right mouse peduncle, illustrating the origin of infrasubthalamic collaterals from its middle sector (i.e., the pyramidal tract component). Note some of the fibers appear to bifurcate shortly after their origin.

According to Cajal, these fibers arise clearly as collaterals of pyramidal tract fibers coursing through the middle sector of the peduncle (Figures 2A,B; this is the known topography of motor cortex pyramidal tract efferents; Déjerine, 1895). They course caudalwards within the diencephalic prerubral tegmentum, passing deep to the substantia nigra (whose compact part is partly crossed: Figure 1C) and underneath or partly through the zona incerta (Figures 1A–C). The ifsth-py-coll fibers then continue caudalwards under the thalamus and pretectum, until they are lost in the neighborhood of the rubral capsule (Figures 1A–C). The red nucleus referred to apparently was the parvicellular part, now ascribed to the pretectal tegmentum within the prosomeric model (Puelles, 2013, 2016, 2019). Ramón y Cajal conjectured possible endings of the ifsth-py-coll tract either in the red nucleus, or more caudally in the brainstem; he also considered the spinal cord as a possible long-range target but could not resolve this point by descriptive analysis.

Once the authentic pallido-thalamic nature of the true lenticular fascicle of Forel was ascertained experimentally, first with the Marchi method (Wilson, 1914; Ranson and Ranson, 1939; Ranson et al., 1941) and then with reduced silver procedures derived from the Nauta and Gygax method (Nauta and Mehler, 1966; Nauta, 1993), Ramón y Cajal’s ifsth-py-coll tract simply disappeared from the literature. Nobody seems to have been curious about the real nature and target of the peduncular fibers repeatedly illustrated by Ramón y Cajal in different mammals. These were likely to exist, given the expertise of the observer and the abundance of consistent recorded data, although the tract was not what he thought. Probably, they were explained away by his colleagues as a possible component of the pyramidal corticonuclear collaterals. In any case, modern accounts of the pyramidal tract generally do not show or mention Ramón y Cajal’s hypothalamic collateral fibers; the only modern reference to them I am aware of appears in our book chapter on the mouse hypothalamus (Puelles et al., 2012a, p. 299). Interestingly, an *unidentified* apparent peduncular collateral tract duplicating precisely Ramón y Cajal’s description is visible in the sagittal plates 117–112 of the mouse stereotaxic atlas of Paxinos and Franklin (2013). Ramón y Cajal’s tract is particularly distinct in the acetylcholinesterase-stained sections, where they stand out unstained between the positive subthalamic nucleus and compact substantia nigra formations.

### Assessing the problem within the prosomeric model

Ramón y Cajal’s neuroanatomic background is strictly unknown (he barely touched this topic in his “Recollections of my Life”; Ramón y Cajal, 1923), but his drawings and citations suggest he basically followed Forel (1877, 1907), Déjerine (1895), Von Köllicker (1902), van Gehuchten (1906), and His (1904). He never referred to Herrick’s (1910) and Kuhlenbeck’s (1927) columnar model, nor to the alternative neuromeric models (e.g., Orr, 1887; von Kupffer, 1906).

My analysis below employs an updated neuromeric schema, the prosomeric model (see review in Puelles, 2024), whose *forebrain axis* (when traced along the alar-basal boundary) closely approximates that of His (1904) (probably used also by Ramón y Cajal). In this model, the diencephalon proper lies *caudal* to the hypothalamo-telencephalic complex or secondary prosencephalon, and its alar and basal parts lie rostral to the midbrain. It subdivides anteroposteriorly into three diencephalic prosomeres p1–p3, whose alar domains represent the pretectum (alar p1), thalamus (alar p2), and prethalamus (alar p3). The corresponding three basal (tegmental) domains are referred to roughly in the literature as ‘prerubral tegmentum’. This domain is largely occupied by the diencephalic extension of the mesodiencephalic substantia nigra and the ventral tegmental area, apart some deep preoculomotor reticular cell populations and the nucleus of Darkschewitsch. The red nucleus (nucleus ruber) is classically divided into a caudal magnocellular part, which lies in the midbrain tegmentum (basal m1), and a rostral parvicellular part found in the pretectal tegmentum (basal p1); thus, strictly, the prerubral tegmentum should be ascribed only to the p2 and p3 basal plate and floor.

The prosomeric model divides the hypothalamus into rostral terminal and caudal peduncular parts (THy, PHy); these participate, respectively, in two hypothalamo-telencephalic prosomeres (PHy in h1 and THy in h2; both hypothalamic parts have alar and basal regions; the overlying telencephalon is entirely alar; review in Puelles et al., 2012a; Puelles and Rubenstein, 2003, 2015; Puelles, 2018; Puelles et al., 2023; Diaz et al., 2023; Puelles, 2024). The subthalamic nucleus (STh) is presently understood as a basal hypothalamic cell population of h1 (PHy) that originates in the retromamillary area (RM; review in Puelles et al., 2012a; Puelles, 2019; López-González et al., 2021). During the development, the STh neurons migrate *subpially* dorsalward within PHy, stopping at a more dorsal locus within the local hypothalamic retrotuberal basal plate. The STh primordium results covered thereafter by the cerebral peduncle that descends dorsoventrally from the telencephalon via PHy, superficially to the lateral hypothalamus. Once it descends past the STh, the peduncle visibly bends caudalward into the diencephalon, continuing thereafter in a longitudinal tegmental course through the diencephalon and midbrain into the brainstem and spinal cord.

Ramón y Cajal’s infrasubthalamic pyramidal collateral fibers would thus emerge from the caudal aspect of the peduncle ventrally to the migrated subthalamic nucleus, just before the peduncle bends into the diencephalic tegmentum. The ifsth-py-coll fibers would thus penetrate likewise longitudinally the diencephalic tegmentum (Figure 1) but coursing instead deeply in contrast to the subpial peduncle and the associated substantia nigra. They reportedly proceed into the rubral tegmentum (basal p1 and m1) under the zona incerta. In this course, Ramón y Cajal’s fibers would be covered first by the prethalamic alar zona incerta (Puelles et al., 2021; Diaz et al., 2023) and thereafter by the thalamic alar subparafascicular nucleus. It should be noted that it was not clear to anybody at Ramón y Cajal’s time, nor possibly to some present-day neuroanatomists, that the *zona incerta* (ZI) is a ventral *prethalamic alar plate derivative* (see genoarchitectonic evidence in Puelles et al., 2021). Moreover, at the time, it was not known either that large rostral parts of the substantia nigra and ventral tegmental area of Tsai are diencephalic tegmental rather than mesencephalic tegmental (see Puelles and Medina, 1994; Puelles et al., 2012a; Puelles, 2013; Puelles, 2019). It has to be admitted that the prerubral tegmentum with its fields of Forel and nigral cell populations is all round not the best-understood part of the brain, although it was one of the first parts of the diencephalon studied (Forel, 1877).

### Poorness of literature on pyramidal collaterals

Literature discussions on extratelencephalic pyramidal tract collaterals largely refer to distinct corticonuclear, corticopontine, and corticospinal components, sometimes mentioning other possible collaterals targeting the red nucleus, or the reticular formation (e.g., Brodal, 1981; p. 187). However, in an ample review of neuroanatomic literature, I was surprised to find that precise indications of *where exactly such* var*ious collaterals arise from the pyramidal tract* and their detailed course into their targets is not described at all in either older or modern neuroanatomic treatises. The schemata provided by the Allen Institute are highly speculative and do not include Ramón y Cajal’s ifsth-py-coll elements.

I include myself in this criticism since I taught human neuroanatomy to medical students for 46 years (1972–2018); over these years, I perused all neuroanatomy books that fell in my hands (in Spanish, English, French, German, Italian, and Portuguese) and never wondered about lack of data on this point. In contrast, every time I leafed through the second volume of Ramón y Cajal’s major treatise (Ramón y Cajal, 1911), or his book on diencephalic studies (Ramón y Cajal, 1966), I was invariably attracted to his observations on ‘Forel’s lenticular fascicle’, knowing that this interpretation of Ramón y Cajal was contradicted by all modern notions on pallido-thalamic connections. I simply could not believe that Ramón y Cajal was inaccurate to the point of drawing an inexistent tract, which he actually saw with both Golgi and Ehrlich methods in a handful of mammalian species. His precise drawings of the ifsth-py-coll fibers seem absolutely credible, irrespective of the remarkable silence about them in the experimental or textbook literature on motor cortical projections over these last 100 years or so.

This clearly forgotten centenary issue may benefit from being reexamined under a new perspective, starting with the terminological change I propose within the updated prosomeric model. I have renamed Ramón y Cajal’s ‘lenticular peduncular fibers’ arising at basal plate levels ‘*infrasubthalamic pyramidal collaterals*’ (ifsth-py-coll). Other caudally coursing hypothalamic pyramidal collaterals arise at (suprasubthalamic) alar plate levels of the peduncular hypothalamus (e.g., *thalamic, entopeduncular,* and *subthalamic* collaterals). The hypothalamic origin is certain, in any case, since the collaterals emerge in sagittal sections just *under* the subthalamic nucleus (STh), before the peduncle bends (Puelles et al., 2012a; Puelles and Rubenstein, 2015; Puelles et al., 2021, 2023; Diaz et al., 2023; Paxinos and Franklin, 2013, plates 112–117; see Figure 1).

### The present approach

The availability of a sizeable set of modern experimental hodologic experiments in the mouse brain, whose results are offered publicly to the scientific community by the Allen Institute for Brain Science in a database named Mouse Brain Connectivity(see text footnote 1), suggested the possibility to check Ramón y Cajal’s description of the ifsth-py-coll tract. The cited database includes numerous experiments restricted to the primary and secondary motor cortex, as well as others labeling alternatively the nearby somatosensory or cingulate ‘motor’ cortex areas, where some descending fibers of the pyramidal tract also might originate. Under optimal conditions, such hodologic material should verify the ifsth-py-coll fibers and illuminate where the terminals of such collaterals end.

I found indeed that numerous Allen Mouse Brain Connectivity experiments consistently corroborate the existence in the mouse of Ramón y Cajal’s ifsth-py-coll tract, exactly as described by him, with some unexpected additional details (see below). I soon found out that these fibers do not target the red nucleus, or any other more caudal brainstem site originally considered by Ramón y Cajal. The fibers rather separate into deep periventricular and outer (lateral) tegmental components. The deep fibers apparently project *en passant* upon the deep zona incerta and thereafter terminate sequentially on thalamic, pretectal, and midbrain periventricular sites possibly associated with preoculomotor functions. The outer descending ifsth-py-coll fibers, the main component, gradually seem to pass from the prerubral tegmentum into the overlying thalamic and pretectal alar plate. When they penetrate the midbrain tectal territory, they suddenly diverge radially pialwards toward a massive terminal ending at a superficial *paratectal* alar midbrain locus adjacent to the tectal gray and the superior colliculus. These outer fibers, which seem to be those depicted by Ramón y Cajal, possibly contact *en passant* other prethalamic, thalamic, and pretectal alar centers. The terminal paratectal griseum is badly understood in conventional neuroanatomic literature or rodent brain atlases, where it is often misinterpreted as a lateral part of the middle stratum of the superior colliculus, which is aberrantly devoid of a covering optic fiber layer and a superficial stratum (see reviews in Puelles E. et al., 2012; Puelles, 2019, 2021; it was argued that this interpretation is contradictory with what we know of tectal development and evolution). In the ‘Midbrain’ chapter of the Mouse Nervous System book (Puelles E. et al., 2012), we renamed this paratectal griseum the *bed nucleus of the brachium of the superior colliculus* (BSC); indeed, it coincides topographically with that subpial tract and lies dorsally to the BIC nucleus (BSC; BIC; Figure 1C). Benavidez et al. (2021; see Discussion) recently established that this paratectal center receives pyramidal afferents, although these authors interpreted the locus as part of the superior colliculus.

In summary, data mining in a rich public database of mouse brain connections corroborates the real existence of Ramón y Cajal’s *ifsth-py-coll tract* and identifies it as a motor pathway targeting visuomotor incertal, pretectal, paratectal, and preoculomotor neurons, possibly conveying *cortical collateral-copy motor signals* significant for gaze control.

These findings are presented within the morphologic framework of the updated prosomeric model (Puelles, 2013; Puelles, 2019; Puelles et al., 2012a, 2013;^39,19^; Puelles and Rubenstein, 2015; Ferran et al., 2015; Garcia-Calero et al., 2020; Puelles et al., 2001, 2016, 2023; Puelles, 2018, 2019, 2021, 2024). This paradigm allows a topologically precise genoarchitectural (eventually causal) characterization of neuroanatomic brain regions and axonal courses (Puelles, 2024).

## Materials and methods

**Figure 3:**
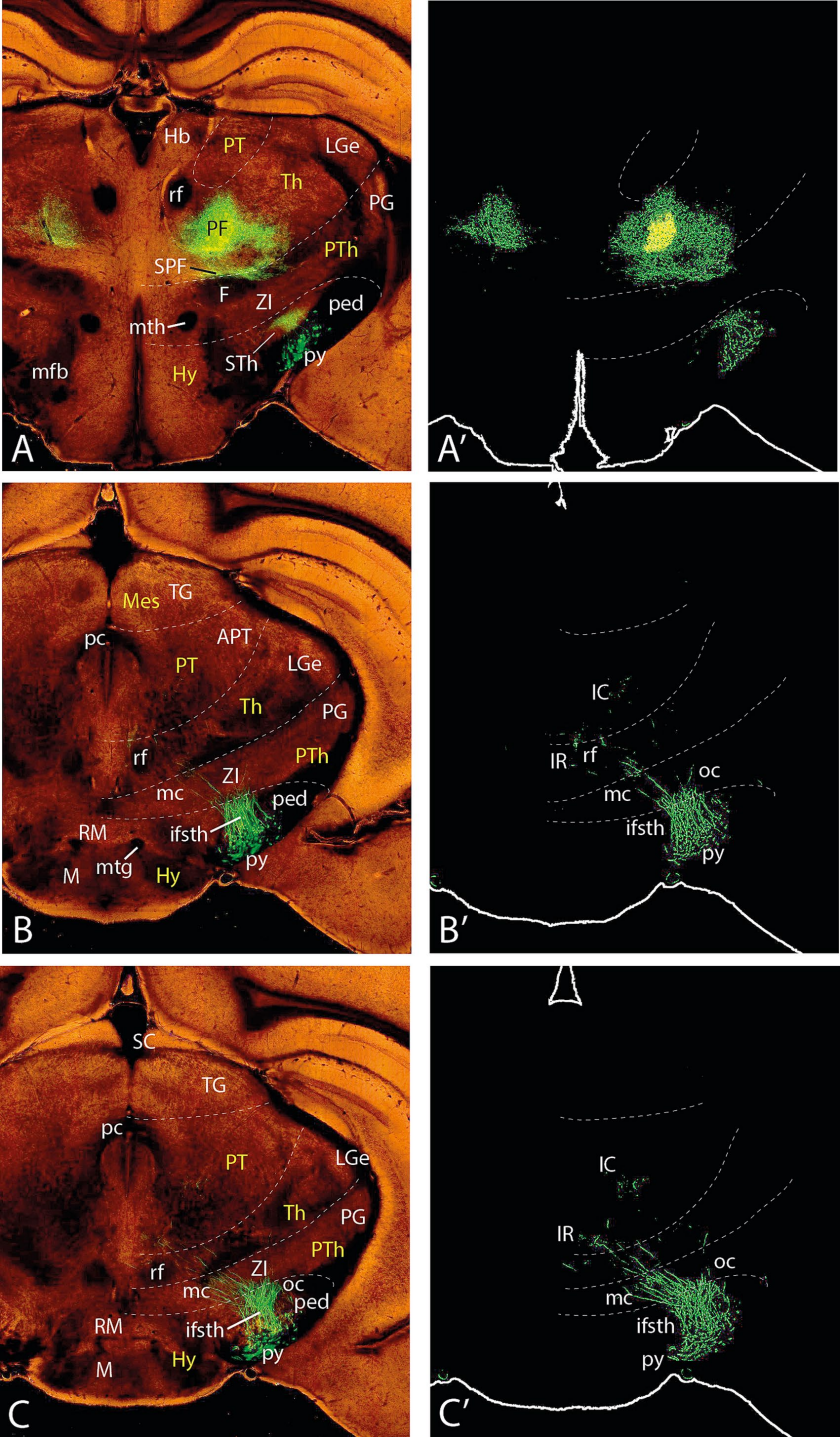
Topologically horizontal sections (experiment MOp-MOs 272,697,944) showing the course and terminals of the ifsth-py-coll tract between hypothalamus and midbrain. **(A, A**′**)** illustrate a section level through the parafascicular and subthalamic nuclei (labeled independently from the ifsth-py-coll tract). **(B–C′)** Show two sections at the origin of the ifsth-py-coll tract.

**Figure 4:**
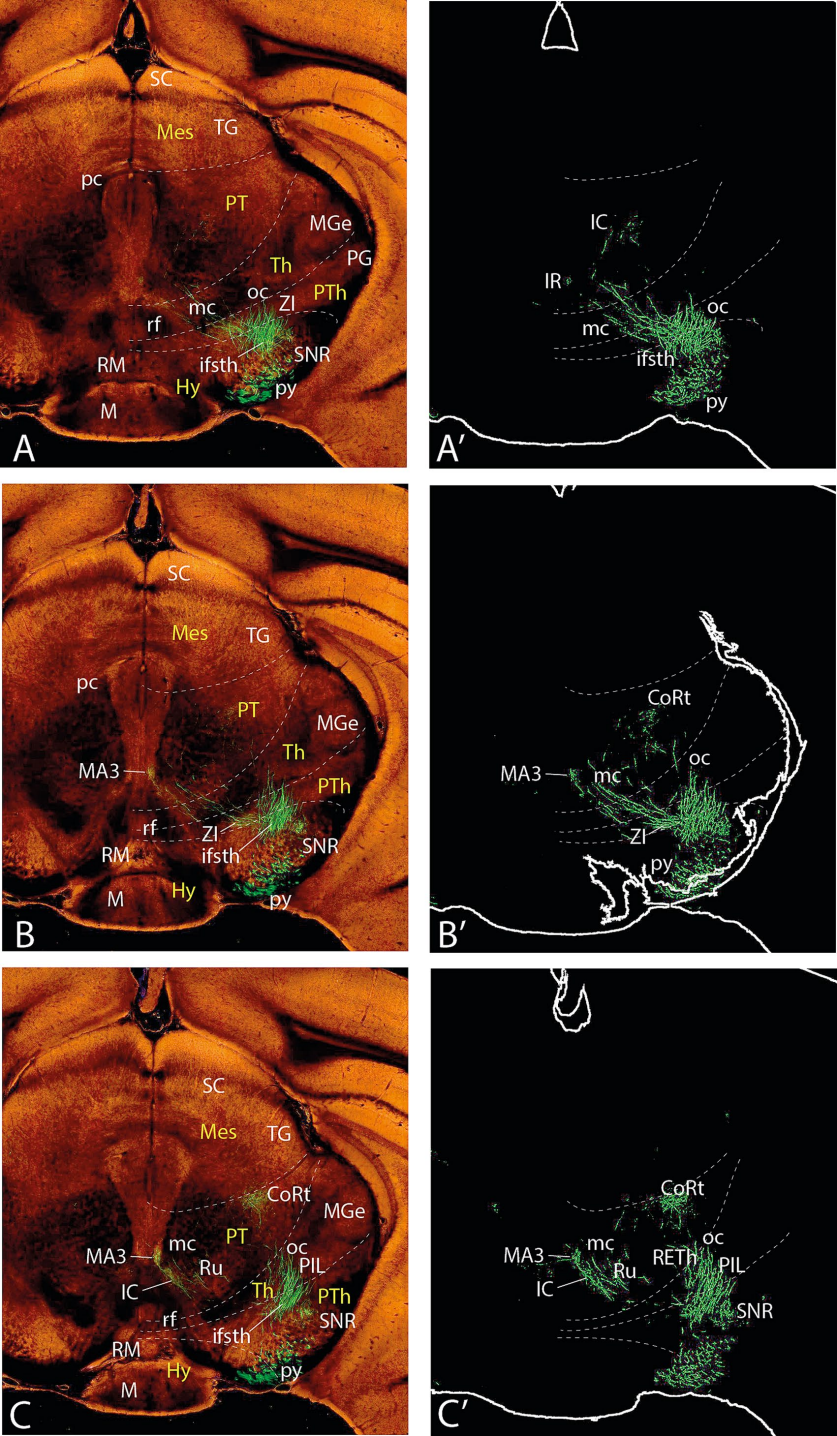
Topologically horizontal sections (experiment MOp-MOs 272697944) showing the course and terminals of the ifsth-py-coll tract between hypothalamus and midbrain. **(A–C′)** The three section levels illustrated interest the deep ifsth-py-coll component traversing the ZI and the field of Forel with the correlative periventricular area. The outer component is also visualized crossing a more lateral part of the ZI and entering the thalamic PIL area.

**Figure 5:**
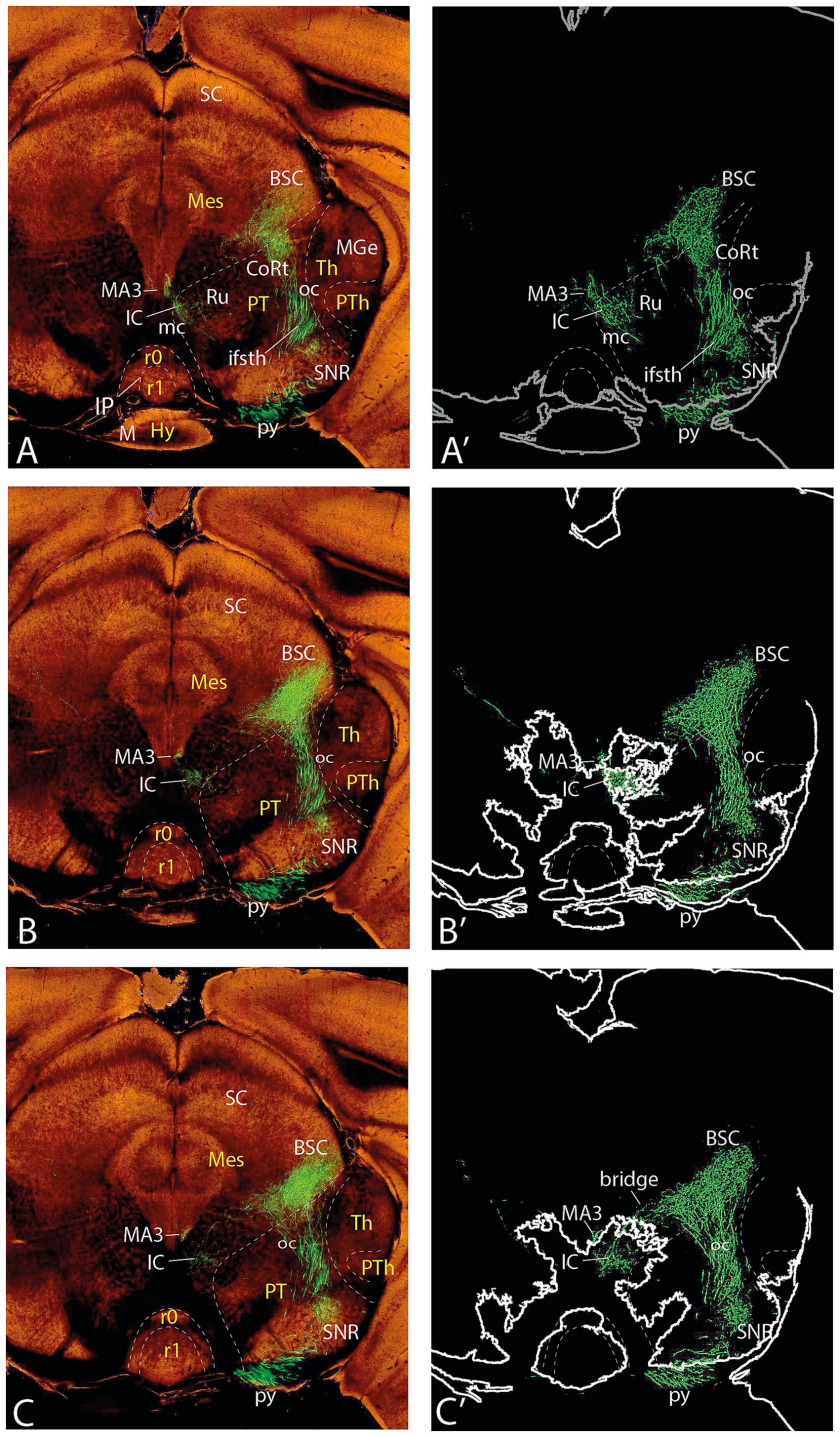
Topologically horizontal sections (experiment MOp-MOs 272697944) showing the course and terminals of the ifsth-py-coll tract between hypothalamus and midbrain. **(A–C′)** The three section levels illustrate the deep ifsth-py-coll component reaching pretectal and midbrain areas just external to the periaqueductal gray (PAG) with the correlative periventricular and interstitial reticular projections. The outer ifsth-py-coll component is also visualized as it crosses successively the thalamic retroethmoid nucleus and the pretectal and midbrain lateral reticular formation (deep to the medial geniculate nucleus, the superficial pretectal formations, and the BIC), finally changing to a radial course into the superficial BSC paratectal nucleus within the midbrain. Note also a projection field in the SNC/SNR.

**Figure 6:**
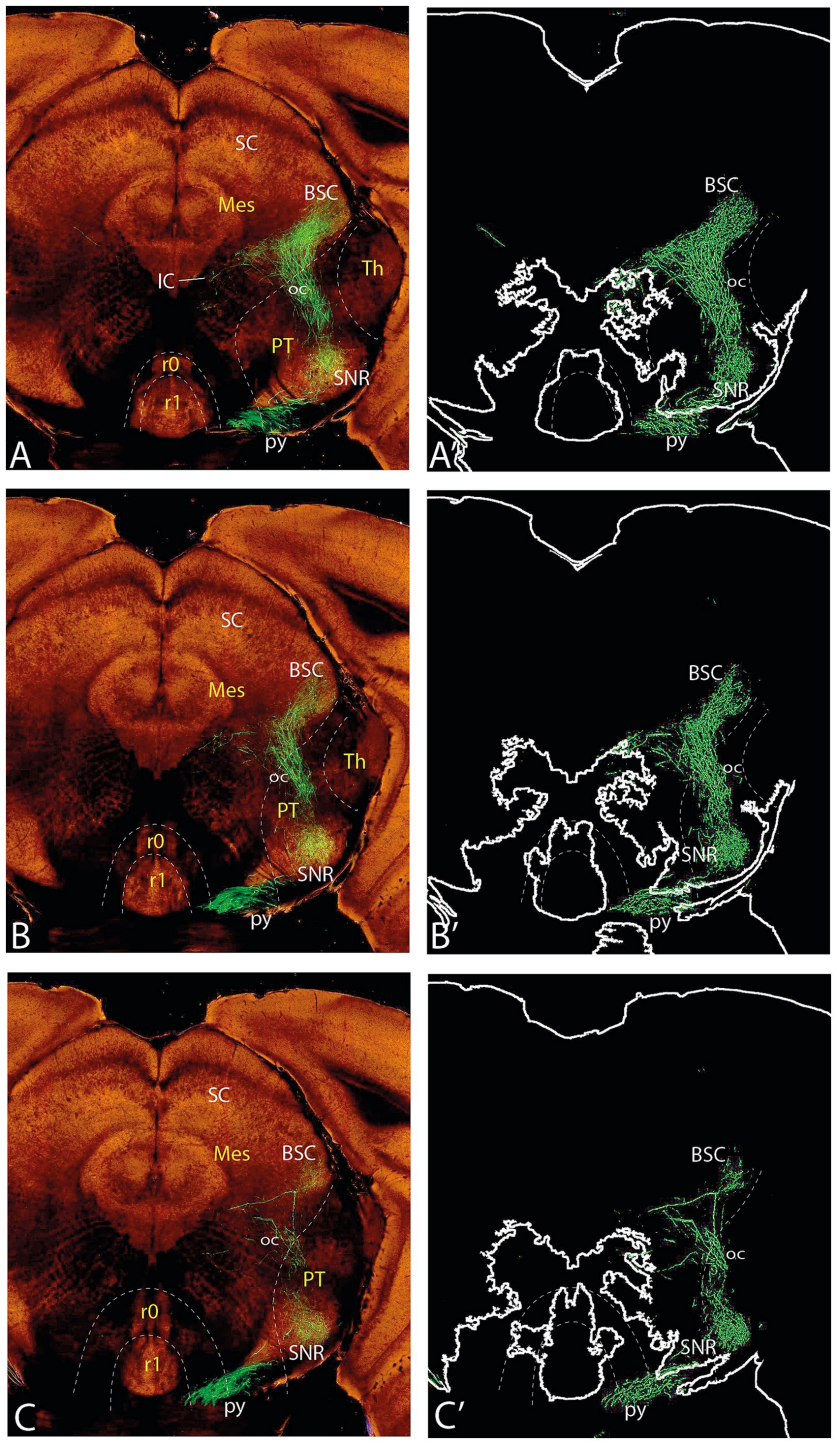
Topologically horizontal sections (experiment MOp-MOs 272697944) showing the course and terminals of the ifsth-py-coll tract between hypothalamus and midbrain. **(A–C′)** These three section levels interest the caudal levels of the deep ifsth-py-coll component distributing to preoculomotor pretectal and midbrain reticular areas external to the periaqueductal gray (PAG), jointly with the correlative outer ifsth-py-coll component visualized as it crosses successively the pretectal and midbrain lateral reticular formation. Note the thalamus is reduced at these levels to a diminishing lateral remnant associated with the medial geniculate nucleus. The neighboring superficial pretectal formations and the BIC are not clearly distinguishable. We see also the caudalmost parts of the superficial BSC paratectal nucleus within the midbrain, still receiving radial terminals from the ifsth-py-coll. Note also the projection field in the SNR.

**Figure 7:**
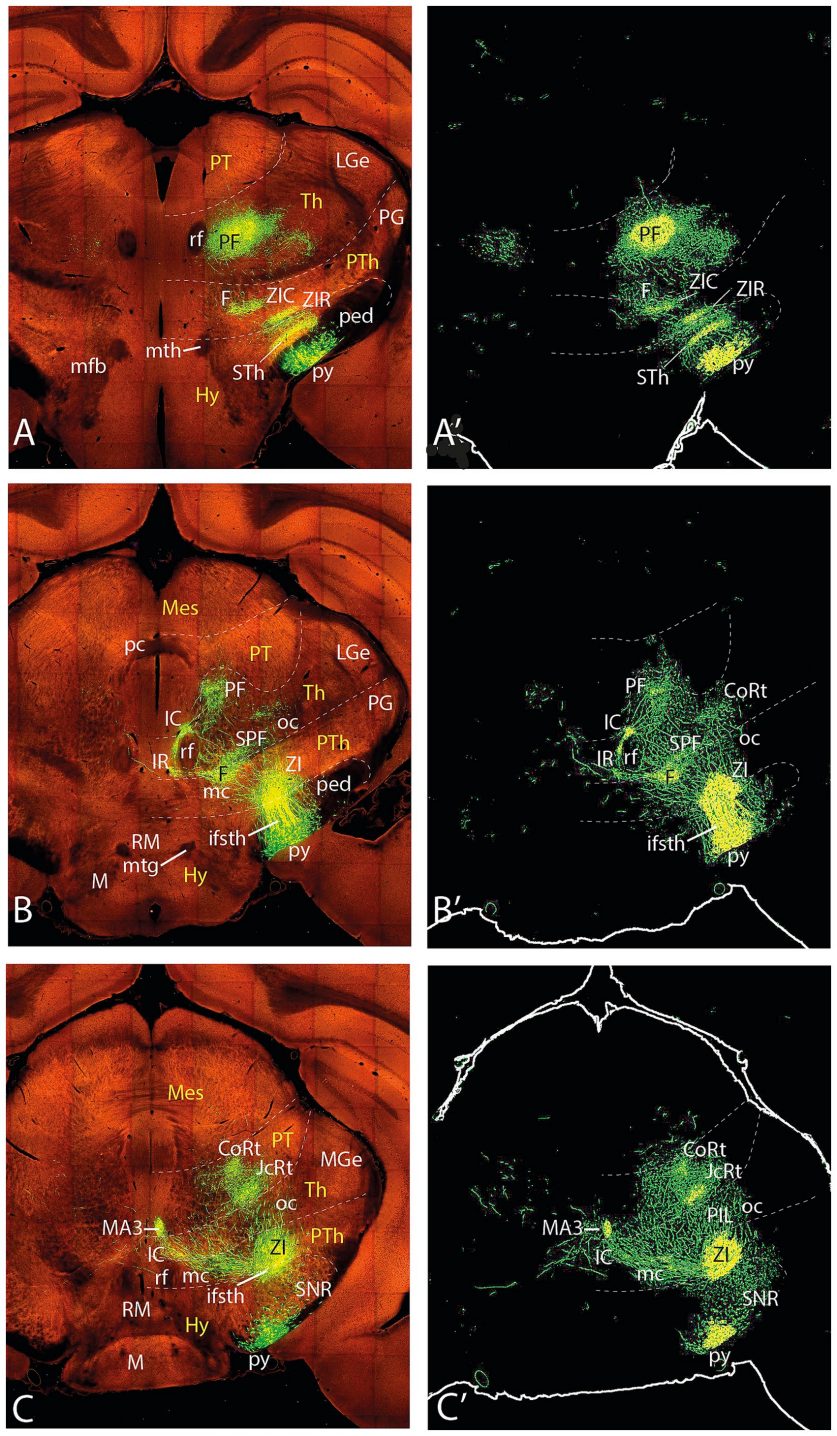
Topologically horizontal sections (experiment MOp 100141780) showing the course and terminals of the ifsth-py-coll tract between hypothalamus and midbrain in experiment MOp 100,141,780 (first level of 5). **(A,A’)** Illustrate a section level above the ifsth-py-coll tract origin, passing through the (labeled) parafascicular nucleus, zona incerta, and the subthalamic nucleus. **(B–C′)** show two more ventral sections at the highest origin of the ifsth-py-coll tract. The initial course of the deep and outer components is seen in **(C,C’)**. Note also in **(C,C’)** distinct outer component terminal fields in the thalamus (PIL) and pretectum (JcRt, CoRt), as well as the presence of transnigral-labeled peduncular collaterals.

**Figure 8:**
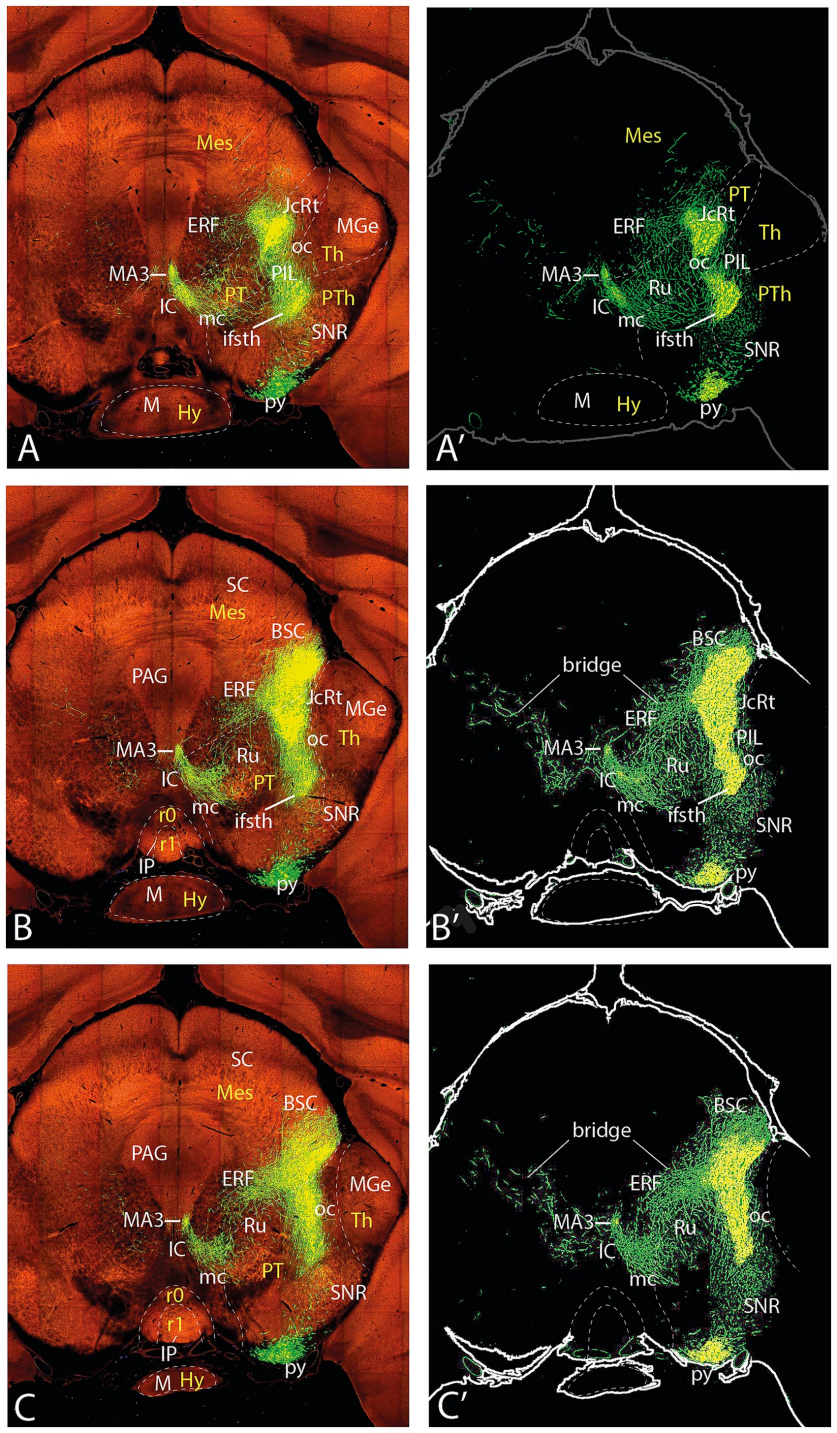
Topologically horizontal sections (experiment MOp 100141780) showing the course and terminals of the ifsth-py-coll tract between hypothalamus and midbrain in experiment MOp 100141780 (second level of 5). **(A,A’)** illustrates a section level that still shows clearly outer component terminal patches in thalamic (PIL, REth) and pretectal (JcRt, CoRt) loci. **(B–C′)** show the arrival of Ramón y Cajal’s tract at the BSC midbrain locus as well as an elaborate deep interstitial (+ MA3) and rubral terminal fields. Transnigral-labeled collaterals are still visible.

**Figure 9:**
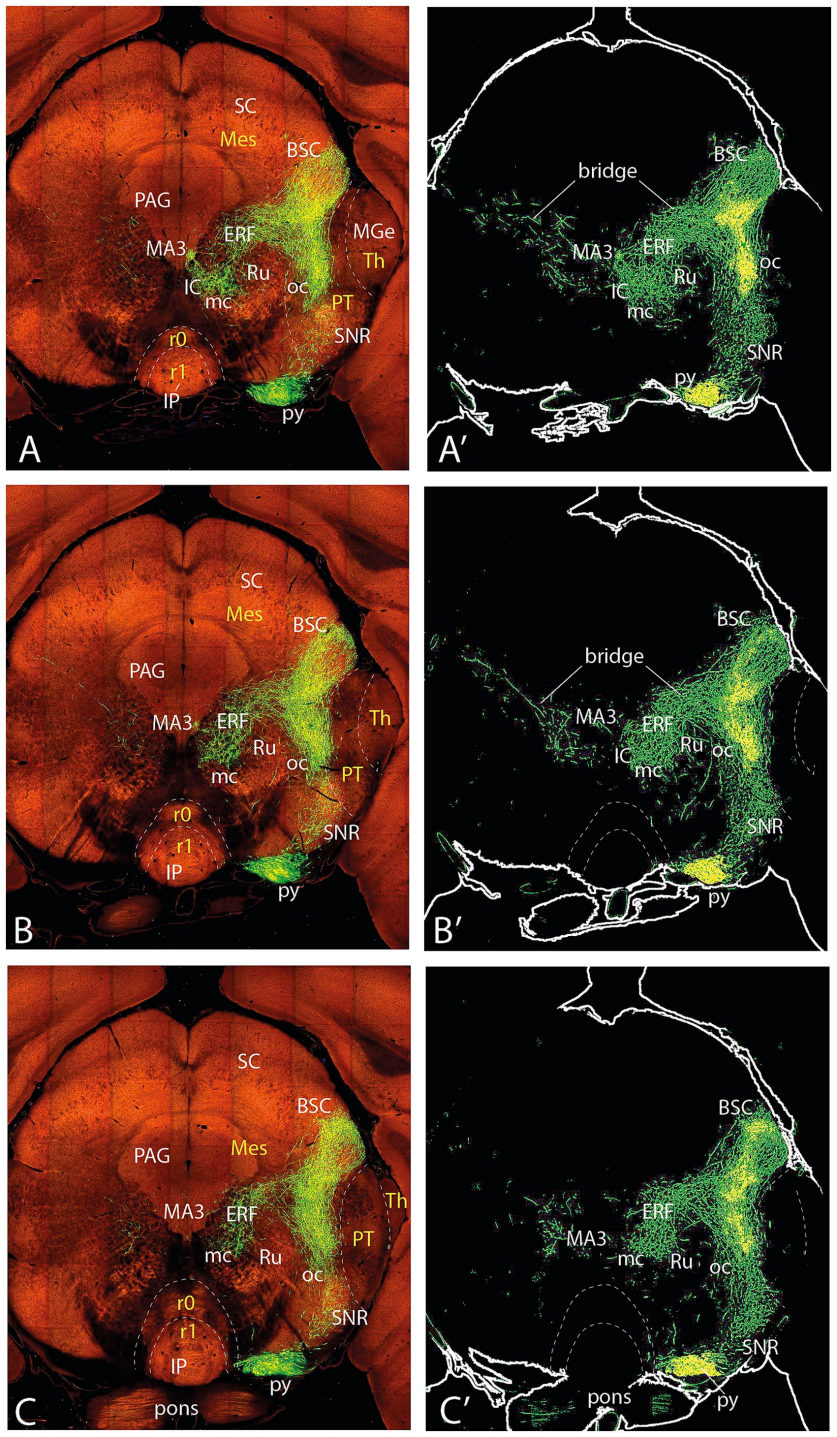
Topologically horizontal sections (experiment MOp 100,141,780) showing the course and terminals of the ifsth-py-coll tract between hypothalamus and midbrain in experiment MOp 100,141,780 (third level of 5). The three section levels **(A–C)** illustrate the BSC terminals and the rubral bridge connecting the deep and outer components of the ifsth-py-coll tract in the midbrain. Transnigral-labeled collaterals are still visible.

**Figure 10:**
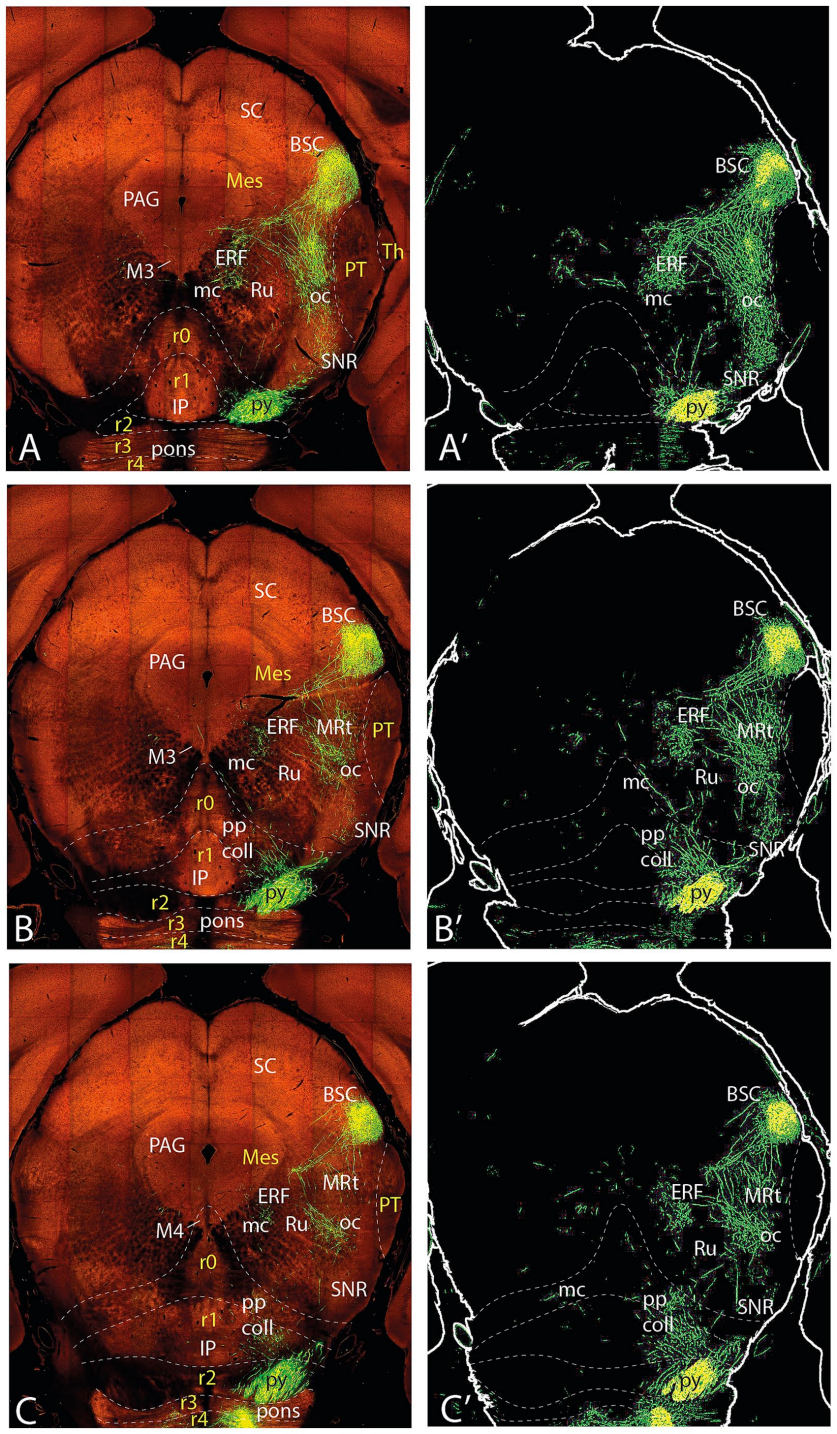
Topologically horizontal sections (experiment MOp 100141780) showing the course and terminals of the ifsth-py-coll tract between hypothalamus and midbrain in experiment MOp 100141780 (fourth level of 5). The three section levels **(A–C)** illustrate the BSC terminals and the gradual end of the diencephalic parts of the deep and outer components of the ifsth-py-coll tract in the midbrain, substituted by terminal deep to the inferior colliculus and within the preisthmic cuneiform nucleus. Transnigral-labeled collaterals gradually end along these three section levels, and another set of pyramidal collaterals emerges laterally to the prepontine interpeduncular nuclear complex, just before the peduncle enters the pons.

**Figure 11:**
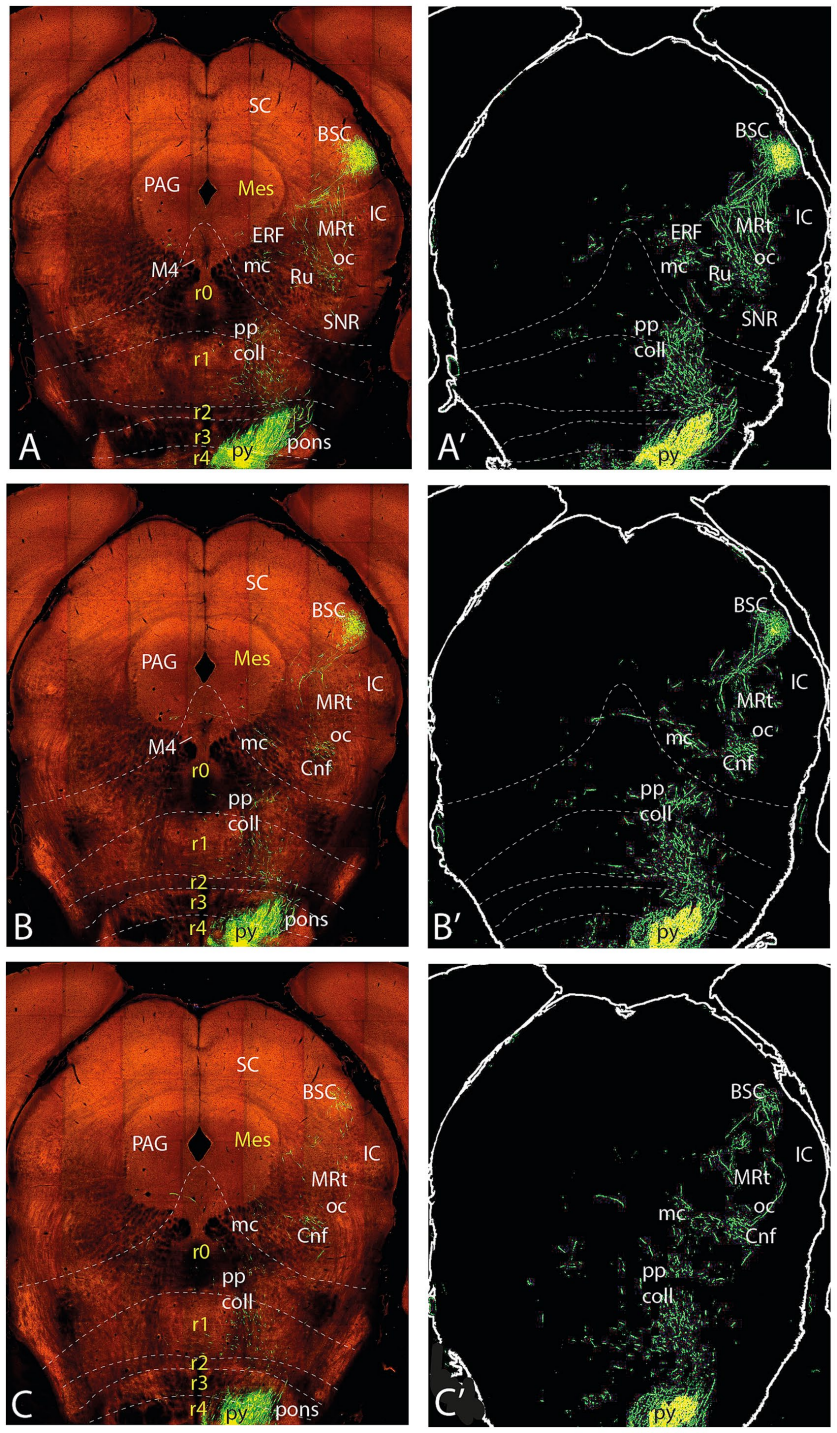
Topologically horizontal sections (experiment MOp 100141780) showing the course and terminals of the ifsth-py-coll tract between hypothalamus and midbrain in experiment MOp 100141780 (fifth level of 5). The three section levels **(A–C)** illustrate the caudalmost BSC terminals (above the IC), the end of mesencephalic terminals of the ifsth-py-coll tract, and the appearance of terminal patches arising from the prepontine collaterals in the isthmus and rhombomere 1.

**Figure 12:**
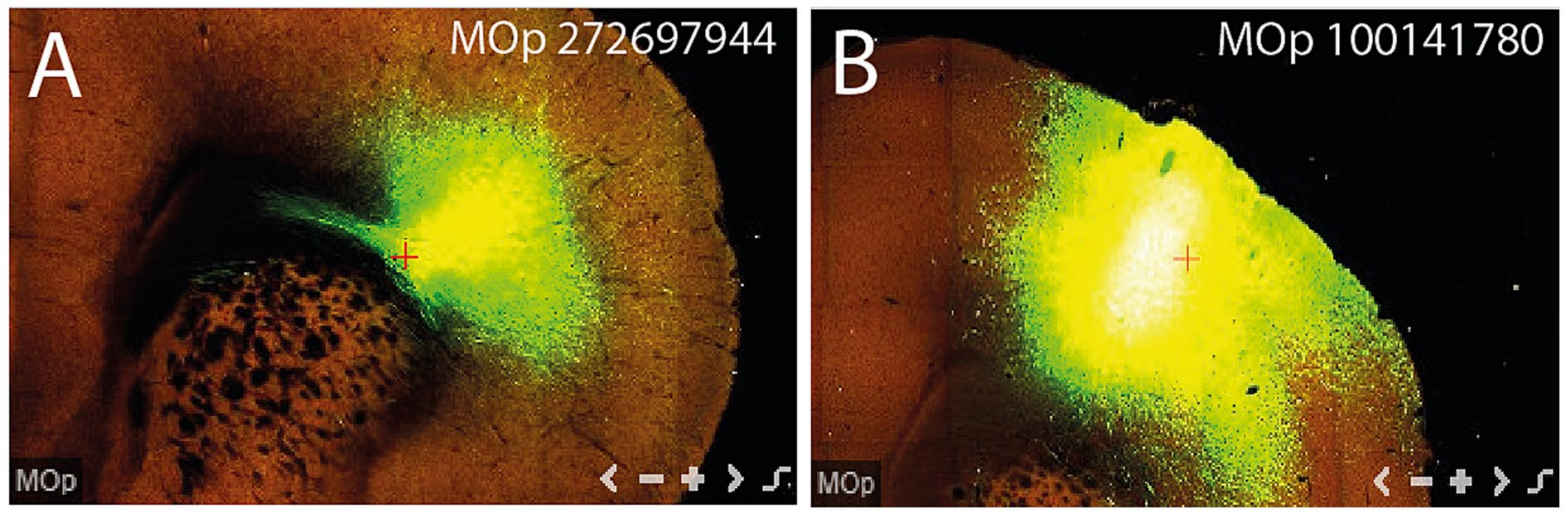
**(A)** Injection site in experiment MOp 272697944. **(B)** Injection site in experiment MOp 100141780.

**Figure 13:**
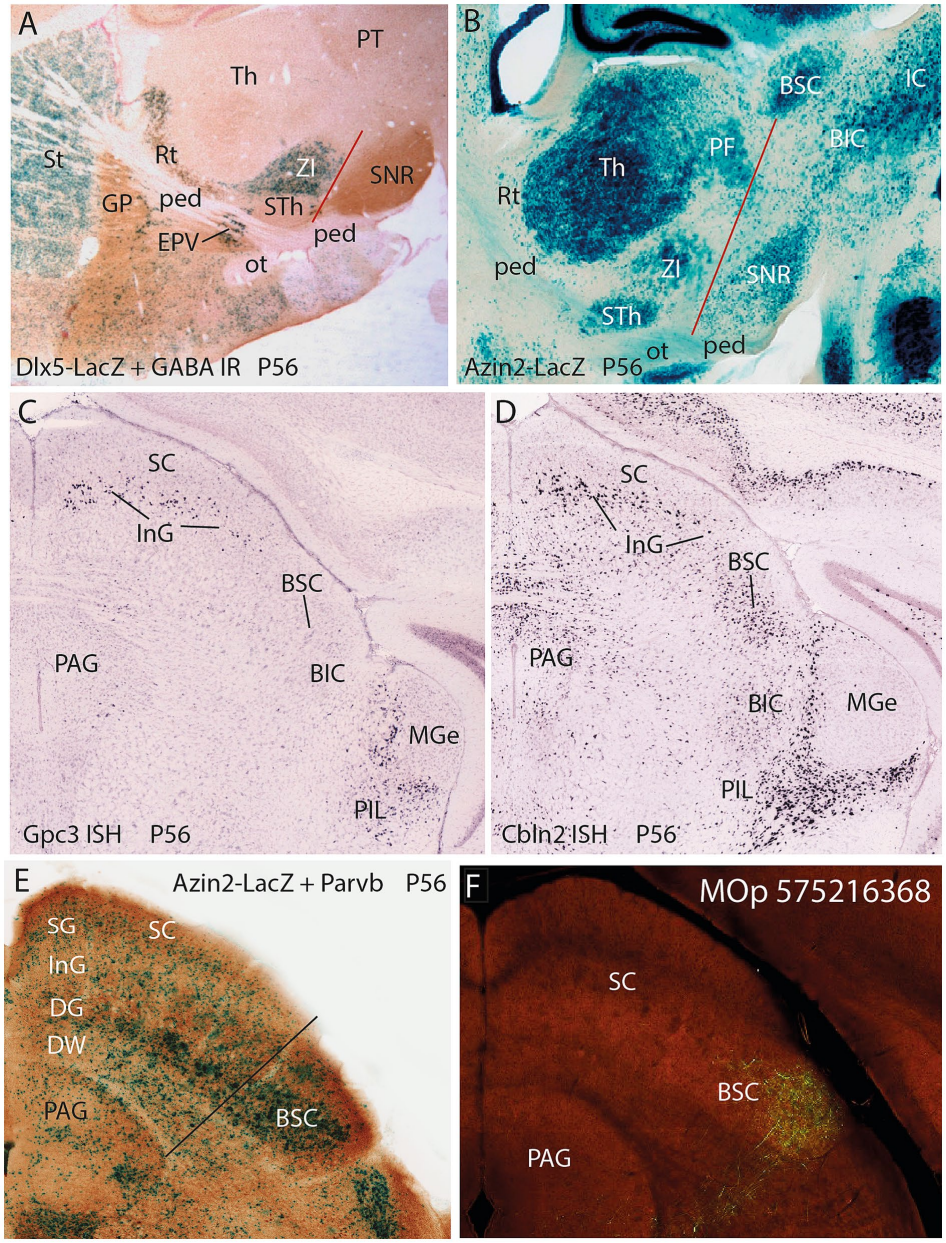
Additional genoarchitectonic images to illustrate the territory related to the ifsth-py-coll tract and its final target, the BSC nucleus in the midbrain. **(A)** Sagittal section of a P56 transgenic mouse reacted for *Dlx5*-LacZ expression (blue reaction product) with additional GABA immunoreaction (brown reaction). Strong *Dlx5* signal appears in the striatum (St), the ventral entopeduncular nucleus (EPV), and the zona incerta (ZI). The basal forebrain including the globus pallidus (GP) and the reticular part of the substantia nigra (SNR) react significantly for GABA. The subthalamic nucleus shows no *Dlx5* and nearly background levels of GABA IR. A red line marks the initial course of the ifsth-py-coll tract, as described by Ramón y Cajal. **(B)** Sagittal section of an adult transgenic mouse reacted for *Azin2*-LacZ expression (blue reaction product). The STh, ZI, and SNR formations distinctly express Azin2, similarly as the thalamus complex with the parafascicular nucleus (Th, PF; note the pretectum is relatively unlabeled). The section cuts the lateral part of the tectal plate, wherein we see the IC/BIC complex and the paratectal BSC nucleus (the SC would be more medial; compare **E**). A red line indicates the trajectory of the outer component of the ifsth-py-coll from the hypothalamic basal plate into the alar midbrain BSC. Both **(A)** and **(B)** show how the peduncle bends around the STh nucleus to enter its longitudinal diencephalic tegmental course. **(C,D)** are similar coronal sections through the P56 mouse midbrain reacted, respectively, for *Gpc3* and *Cbln2* ISH (Allen Mouse Brain Atlas). Both markers label distinctively neurons in the intermediate gray layer of the superior colliculus (InG, SC) but do not label at all the BSC **(C)** or label it in a different, diffuse and weaker pattern **(D)**. These observations support that the BSC is not part of the collicular InG stratum but a separate nucleus (apart of the absence of optic fibers and a superficial stratum characteristic of the SC). Both markers also identify the thalamic PIL nucleus. **(E)** Coronal midbrain section through SC and BSC in an adult transgenic mouse reacted for Azin2-LacZ expression (blue reaction product; compare **B**) counterstained with Pvalb immunoreaction. The three main SC strata—SG, InG, and DG—contrast with the simpler nuclear structure and differential Azin2 and Pvalb reaction of the BSC. A black line marks the SC/BSC boundary. **(F)** A similar coronal section through the adult SC and BSC, the latter labeled anterogradely from the primary motor cortex (Allen Mouse Brain Connectivity Map; experiment MOp 575216368).

Materials used in this study are either drawings copied from Ramón y Cajal’s Studies on the Diencephalon (Ramón y Cajal, 1966; reproduced there from earlier publications in 1900, 1903, and 1911) or public material from the Allen Mouse Brain Connectivity database (see text footnote 1). While some 30 connectivity experiments were studied in detail, only four of them [cases MOp-MOs 272697944, MOp 100141780, MOp-MOs 288160135, and MOp 575216368; all of them received EGFP injections] were illustrated in the Figures. In particular, case MOp-MOs 272697944 appears in Figures 3–6 and case MOp 100141780 in Figures 7–11; in these plates, each section level is visualized in a pair of images X and X’ illustrating, respectively, a ‘serial two-photon tomography’ view (less fiber detail but the background anatomy is visible) and a ‘projection segmentation’ view (no background and optimal fiber detail). Figures 12A,B show the respective injection sites. Some additional images included in Figures 1C, 13 originate from the author’s own collection of mouse brain preparations (previously unpublished) or from the Allen Developing Mouse Brain Atlas.^2^

## Results

**Figure 14:**
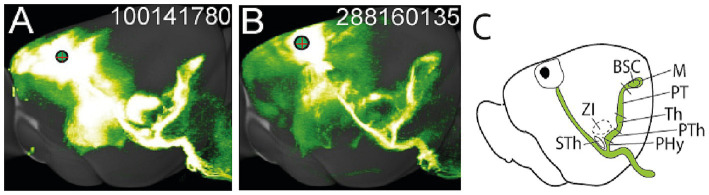
**(A,B)** Allen 3D reconstructions of two cases (code at upper right corner) with EGFP injections (volumes, respectively, of 0.175 and 0.316 microliters) at the primary motor cortex, illustrating the injection site (circle with plus sign; circle proportional in size to the injection), the general descending course of the pyramidal tract, and the ifsth-py-coll tract progressing orthogonally to its infrasubthalamic origin (also under the zona incerta) through the basal and alar diencephalon into the paratectal BSC site in the midbrain tectum (compare Figure 1C). These are two representative cases among some 30 experiments examined in detail. Note the reconstructed shape of the collateral tract is essentially the same in both cases. **(C)** Drawing indicating approximately the prosomeric sectors traversed along the course of the ifsth-py-coll tract.

There is massive and consistent evidence at the Allen Mouse Brain Connectivity database that the pyramidal tract component of the peduncle (its middle part; see Figure 2B), which is labeled from multiple injection sites in the primary motor cortex, indeed releases a packet of hypothalamic infrasubthalamic collaterals. In sagittal sections, this tract looks exactly as reported by Ramón y Cajal (Figures 1A–C; see also Paxinos and Franklin, 2013; their Plates 112–117). The transparentized lateral 3D reconstructions of the individual pyramidal tract experiments offered at the Allen Mouse Brain Connectivity database usually show it distinctly (e.g., Figures 14A–C).

### Experiment MOp-MOs 272697944

#### The telencephalic course

The following description is based on experiment MOp-MOs 272697944 (C57BL/6 J; 0.371 μL EGFP injection) located in the motor face area, close to the boundary with the somatosensory area. The injection site is centered upon layers 5 and 6 (Figure 12). The labeled packet of fibers in the white matter first releases medial callosal projections, which cross the corpus callosum as a compact bundle placed shortly behind the callosal genu (as seen in sagittal reconstruction). The pyramidal tract crosses the striatum and the globus pallidus via the internal capsule (not shown); corticostriatal projections were observed to terminate in the lateral part of the striatum, next to the external capsule.

#### The hypothalamic course

As soon as the tract sorts out of the telencephalon, it initiates its dorsoventral peduncular hypothalamic course (this refers to the axis of the prosomeric model, ending in the hypothalamic postoptic recess, similarly as the classic axis of His (1895), His (1904). The tract first traverses dorsoventrally the alar peduncular hypothalamus (PHy; hypothalamic part of prosomere hp1), crossing successively its longitudinal paraventricular and subparaventricular areas, superficially to the alar lateral hypothalamus and its interstitial medial forebrain bundle. Afterward, it enters the basal retrotuberal region of PHy that contains just deep to the peduncle the migrated subthalamic nucleus (ped; STh; Figure 1C). At the upper alar level of this course, the tract releases orthogonally backwards its transthalamic fibers that proceed caudalwards, crossing anteroposteriorly the prethalamic *reticular nucleus* (where terminals are observed) and entering next the thalamus (some of these fibers reportedly reach the superior colliculus, though none such appeared labeled in this experiment). The thalamic terminals invade ipsi- and contralaterally a sizeable territory that crosses the fused thalamic midline through *the rhomboid nucleus* and extends (more massively ipsilaterally) through *the rhomboid, submedius, ventromedial, and ventrolateral nuclei* and they likewise invade the caudoventral *parafascicular and subparafascicular nuclei* (Figures 3A,A’). There are no other pyramidal collaterals at levels through the alar PHy.

When the pyramidal tract enters the basal PHy, it first relates intimately with the *subthalamic nucleus*, where it generates a dense terminal field (Figures 3A,A’; cortico-subthalamic connection). The labeled pyramidal tract then continues ventralwards past the subthalamic nucleus. It is here, before it bends caudalwards into the diencephalic tegmentum, that it releases orthogonally *Ramón y Cajal’s ifsth-py-collateral tract* (Figures 3B–C’).

The labeled collateral tract enters caudalwards the interstice between the overlying prethalamic zona incerta (subcentral part of the alar prethalamus; Puelles et al., 2021; see Figure 1C) and the underlying prethalamic (rostral) part of the substantia nigra, thus initiating a longitudinal diencephalic tegmental course across the prethalamus, thalamus, and pretectum, that ends at the paratectal BSC site in the rostral midbrain (Figures 14A–C).

#### The diencephalic course

Ramón y Cajal’s infrasubthalamic collaterals penetrate caudalwards the prethalamic tegmentum, possibly also entering partially the lower part of ZI close to the alar-basal boundary. At this place, many of its fibers bifurcate (as was suggested in Figure 2B) into a deep periventricular component carrying relatively fewer fibers (these apparently were not seen by Ramón y Cajal) and an outer richer component that clearly represents Ramón y Cajal’s collateral tract. Both course caudalwards into the midbrain, thus crossing longitudinally the entire diencephalon, all the while entering increasingly the alar region (see Figures 4–6).

The deep component—this component diverges into the deep prethalamic prerubral tegmentum, adopting a position just outside the local periventricular gray, where it releases terminals that invade neighboring *deep parts of the prethalamic zona incerta* and *Forel’s field* (Figure 4). More caudally, they innervate in the thalamic deep tegmental region the *rostral interstitial nucleus of Ramón y Cajal* (Figure 5; this is a thalamic tegmental reticular group of preoculomotor cells lying in front of the retroflex tract, whose axons apparently project to oculomotor motoneurons via the medial longitudinal fasciculus or MLF). At pretectal levels, the deep projection is found at the *interstitial nucleus of Ramón y Cajal* (a pretectal group of preoculomotor reticular neurons that also send descending axons through the neighboring MLF; these reach even the spinal cord via the interstitio-spinal tract). Some terminals seem to converge on the *pre-Edinger-Westphal nucleus* (pEW), a paramedian longitudinal column of neurons with descending axons that reach the spinal cord (Figures 5, 6; see Puelles et al., 2001).

The outer, main component—Coming out of the bifurcation, the majority of outer fibers form Ramón y Cajal’s ifsth-py-coll tract (Figures 4–6), which initially advances caudalwards in the prerubral interstice separating the zona incerta from the substantia nigra compacta, laterally to the nigrostriatal tract. However, the fibers gradually enter alar plate diencephalic domains, first entering partly the overlying prethalamic *ventral zona incerta* (rostral and caudal parts; ZIR, ZIC). Afterward, they cross the ventral part of the thalamic alar territory, which largely corresponds in rodent atlases (e.g., Paxinos and Franklin, 2013) to the *retroethmoid* and *posterior intralaminar* nuclei. Thereafter, they continue ascending straightforwardly through alar pretectal territory forming the *pretectal reticular formation* (there seem to be separate terminal fields in its juxtacommissural and commissural sectors). The tract finally reaches the midbrain lateral reticular formation after passing deep to the brachium of the inferior colliculus and apparently sending some terminals into the subpial *dorsal terminal nucleus*.

#### The mesencephalic end of the ifsth-py-coll course

The outer ifsth-py-coll component thus reaches the lateral part of the midbrain rostral alar plate. This is mainly occupied rostrally by the distinctively *calretinin*- and *netrin-G2*-positive and stratified, retinorecipient tectal gray formation (TG; note this griseum lies clearly caudal to the posterior commissure—the end of the pretectum defined molecularly by *Pax6* expression; unfortunately, the TG appears misidentified as ‘posterior pretectal nucleus’ in most rodent literature; for example, Paxinos and Franklin (2013) have PPT instead of TG in their Figures 54–57; see Puelles, 2022). More caudally, there appears the largely calretinin-negative superior colliculus (SC) with its characteristic stratification. At the lateral aspect of both TG and SC, there appears subpially, in a paratectal position, the BSC (interstitial nucleus of the brachium of the superior colliculus; Puelles E. et al., 2012; Puelles, 2022). This griseum lies just dorsal to the cytoarchitectonically similar BIC (interstitial nucleus of the brachium of the inferior colliculus; see Figure 1C). This superficial paratectal locus is confusingly characterized in much literature (including rodent brain atlases) as a subpial ‘lateral part of the middle and deep tectal strata’, thus referring implicitly to an aberrant superior collicular region where *the standard optic fiber and superficial collicular strata would be absent*, supposedly representing therefore a *non-retinorecipient midbrain tectal domain* (nobody describes this area exactly in these terms, but that is what is implied by the lack of superficial elements, if it were true). In our chapter on the mouse midbrain in The Mouse Nervous System book (Puelles E. et al., 2012), we discrepated from this incoherent interpretation and reinterpreted this paratectal superficial formation as the *bed nucleus of the brachium of the superior colliculus* (BSC); this name is consistent with Swanson’s (1998) and Paxinos and Franklin’s (2013) mapping of the rat subpial brachium of the superior colliculus (bsc) immediately dorsally to the bed nucleus of the brachium of the inferior colliculus (BIC; see Figure 1 in Puelles, 2022). Figures 13A–F illustrate these diverse badly understood centers; see also TG, SC, BSC, and BIC in Puelles E. et al. (2012), Puelles (2019, 2022), and Puelles and Hidalgo-Sánchez (2023).

The outer ifsth-py-coll component adopts a radial orientation deep to the BSC and thereafter penetrates it radially and terminates densely inside its core, partly innervating also its peripheral shell (Figures 5 and 6). No terminal of the ifsth-py-coll ever enters the retinorecipient superior colliculus, or the tectal gray. The only previous description of this projection of the primary motor cortex is found in Benavidez et al. (2021), who interpreted the BSC as a ‘lateral part of the superior colliculus’ (see Discussion). Shortly before ascending radially pialwards into the BSC, the outer tract component interchanges some fibers across the reticular formation with the midbrain end of the deep ifsth-py-coll component. These may be fibers that were misplaced in the wrong branch at the prethalamic bifurcation and have here a second opportunity to reach their appropriate targets.

## Experiment MOp 100141780

I next describe experiment MOp 100141780 (C57BL/6J; 0.175 μL EGFP injection) located somewhat more rostrodorsal in the motor face area than the previous experiment. The injection site is centered upon layers 4 and 5 (Figure 12B). I will consider only the aspects of the ifsth-py-coll tract that show some differences in this case since the fundamental structure is the same. Curiously, this experiment received about half the volume of injected marker than the previously described case but shows nevertheless more abundant fibers and terminals.

In topologically horizontal sections through the subthalamic nucleus visualizing its labeled terminal field (above the origin of the ifsth-py-coll tract), distinct separate terminal fields are also observed in the ZIR and ZIC areas, plus additional terminals in Forel’s field and the parafascicular nucleus (F, PF; Figure 7A,A’). It is unclear whether these two ZI projections arise from the subthalamic collaterals or from the infrasubthalamic ones. In the next two sections through the ifsth-py-coll origin (Figures 7B–C’), these two ZI terminal fields fuse into a single larger patch of ZI terminals and the Forel field terminals also expand, which possibly suggests their derivation from the ifsth-py-coll. These details associated with the deep component were not distinguished in the previously presented experiment (Figures 3A–C′). Another peculiarity is that the prethalamic and thalamic space that separates the deep and outer ifsth-py-coll tract components appears filled with a network of fine terminals (Figures 7B’,C’). The caudal end of the labeled thalamic parafascicular nucleus is still visible in Figures 7A,A’. The next Figures 7B–C’, 8A,A’ show discrete terminal fields of the outer ifsth-py-coll tract component in the thalamic PIL/REth area, as well as in the juxtacommissural and commissural pretectal reticular areas (JcRt, CoRt), more distinctly than was seen in the previous experiment. The deep component displays in Figures 7B,B’ more numerous terminals in the rostral interstitial nucleus (RI) as well as in Ramón y Cajal’s interstitial nucleus (IC) (extending into Figure 8). Figures 7C,C’, 8A–C’ show dense labeling of what is identified as medial accessory oculomotor nucleus (MA3) in the Paxinos and Franklin (2013) mouse brain atlas, jointly with a fan of more dispersed terminals around cells probably on the IC which spread medially to the red nucleus (Ru), whose field also receives a sparser number of terminals, apparently coming from both deep and outer components (Figures 8A’–C’). Figure 9 suggests that more caudally, the rubral terminals become restricted to a distinct periventricular field deep to the red nucleus proper, which I propose to name the ‘endorubral field’ (ERF). Part of this midbrain deep terminal pattern (including the ERF) is reproduced less markedly contralaterally by fibers crossing the local floor plate (Figures 7–9). The BSC is first reached by ifsth-py-coll terminals of the outer component in Figures 8B–C’. At these levels, the mesencephalic tegmental bridge connecting the caudal ends of the outer and deep components of the ifsth-py-coll pathway appears massively labeled in this experiment (bridge; Figures 8B–C’, 9A–B’). Notably, this bridge seems to represent also a particular terminal patch, possibly the beginning of the ERF (rather than a mere passage of fibers from one component into the other), since it is formed also contralaterally (specularly).

These sections also show abundant pyramidal collaterals apparently sorting out of the peduncle as it descends along the SNR; these penetrate the SNR (in the previously described experiment, a terminal field was seen within SNR), and some of these *transnigral* fibers may even contribute more deeply to the outer ifsth-py-coll component, practically throughout the midbrain (see Figures 7–10A–B’). At caudal midbrain levels, a separate field of terminals emerges ventrally to the BSC, deep to the inferior colliculus (IC) identified here as mesencephalic reticular formation (MRt; IC; Figures 10B–C’, 11). The caudalmost outer fibers apparently generate a distinct terminal patch as well in the topologically *caudal cuneiform nucleus* (Cnf; Figure 11B–C’). The latter forms an intermediate stratum reticular specialization of the preisthmus (m2-derived midbrain; Puelles and Hidalgo-Sánchez, 2023), identified in the physiological literature as the ‘mesencephalic locomotor center’ (MLC; Orlovsky and Shik, 1976; interestingly, a ‘subthalamic locomotor center’ or SLC is also mentioned in this report; present data suggest a connection via the ifsth-py-coll tract between the SLC—in fact the Mop—with the MLC).

In caudal sections beyond the cephalic flexure that show ventrally the prepontine interpeduncular nuclear complex and start to cut through the pons (Figures 9C,C’, 12), new pyramidal collaterals arise roughly at rhombomere 2 level from the deep aspect of the peduncle just before it plunges into the pons. The prepontine collaterals thereafter spread into tegmental fields within isthmus (r0) and rhombomere 1 (pp coll; Figures 10, 11). This origin of collaterals probably lies at rhombomere 2 level since these elements are not present at more rostral (rhombomere 0–1) levels of the interpeduncular complex. The cited caudal midbrain elements gradually end along the section levels in Figure 11, whereas the prepontine collaterals distribute separately within the reticular tegmental fields of the isthmus and rhombomere 1 (see Figure 11).

## Discussion

### Corroboration of Ramón y Cajal’s description

**Figure 15:**
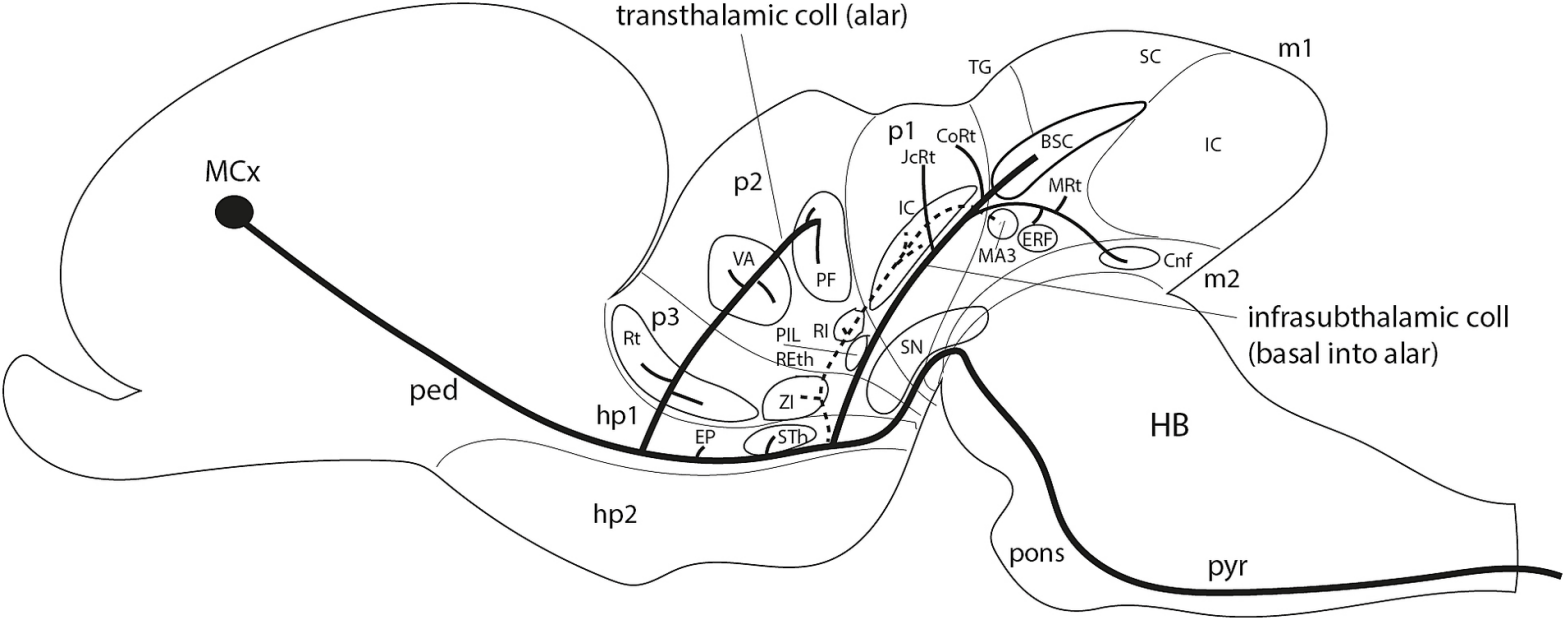
Schematic representation of the infrasubthalamic pyramidal collateral tract of Ramón y Cajal and its connections. The drawing distinguishes the forebrain neuromeric units visualized (thin transverse limits separating hp2, hp1, p3, p2, p1, m1, and m2) plus the hindbrain (HB). The thick line originated in the motor cortex (MCx) is the pyramidal tract coursing into the spinal cord. Along its hypothalamic peduncular course (dorsoventral within the peduncular hypothalamus; ped; hp1 prosomere), it produces first suprasubthalamic transthalamic collaterals at alar plate levels that innervate sequentially the reticular nucleus (prethalamus; p3) and the ventral anterior and parafascicular nuclei (VA; PF; motor thalamus; p2). Other alar collaterals apparently reach the entopeduncular nuclei (EP). The peduncular motor pathway next enters the hypothalamic basal domain, where it first innervates the subthalamic nucleus (STh; basal hp1) and secondly produces the infrasubthalamic pyramidal collaterals of Ramón y Cajal. These fibers penetrate the gap between the zona incerta (ZI) and the substantia nigra (SN) and bifurcate into deep periventricularly coursing (dash line) and more superficial, laterally coursing (continuous black line) components. The deep portion connects with the overlying ZI and some thalamic, pretectal, and midbrain preoculomotor nuclei (RI, IC, and MA3; dash lines). The lateral component initiates an oblique prerubral tegmental course that soon brings them into the thalamic, pretectal, and mesencephalic alar plate. The main terminal target seems to be the paratectal bed nucleus of the brachium of the superior colliculus (BSC; m1), although other terminals are distributed en course to thalamic (PIL; RETh; p2) and pretectal cell populations (JcRt; CoRt; p1), as well as to the midbrain lateral reticular formation (MRt; m1), a novel bilateral endorubral field (ERF; m1) and the preisthmic cuneiform nucleus (Cnf; m2).

The first point to make of these results is that Ramón y Cajal’s description of the ifsth-py-coll tract (under the wrong ‘Forel’s lenticular tract’ name) was absolutely correct. A compact collateral packet of fibers arises out of the pyramidal tract just under the subthalamic nucleus and penetrates caudalwards the interstice between the zona incerta and the substantia nigra (the prerubral diencephalic tegmentum). As concluded by Ramón y Cajal, this tract does not target the thalamus. He missed the conclusion that this tract soon bifurcates into deep periventricular and outer coursing components (Figure 15), although his drawing reproduced in Figure 2B shows a number of bifurcating fibers, attesting to the famed precision with which Ramón y Cajal registered what he saw. It is unclear whether all ifsth-py-coll fibers bifurcate as they enter the diencephalon or not.

The fibers of Forel’s lenticular fascicle, with which Ramón y Cajal confused his infrasubthalamic collaterals, apparently course from the subpallial internal globus pallidus into the motor thalamus in a characteristic perforant pattern, probably crossing the peduncle at an alar level above the subthalamic nucleus, if not through the STh itself (see Puelles et al., 2023). In contrast, according to his drawings, Ramón y Cajal’s ifsth-py-coll fibers clearly sort out of the peduncle *under* the STh, that is, in the hypothalamic basal plate domain (Figures 1A–C, 13A,B, 15 see also Paxinos and Franklin, 2013, Figures 112–117). The fact that the origin of these hypothalamic collaterals was restricted to the middle sector of the peduncle (as seen in coronal sections) allowed Ramón y Cajal to assume their origin in the motor cortex, consistently with Déjerine (1895). The distinctly differential topographic relationship with the subthalamic nucleus of the two sets of fibers (pallido-thalamic versus hypothalamic pyramidal collaterals) was apparently not noticed or attributed to erroneous interpretations by his colleagues by Ramón y Cajal, who thus jumped into his interpretive error.

The apparent reason why the master lost track of the termination of these fibers is that they gradually change their course (thus being cut from the Golgi sections examined), diverging both dorsalward into the alar plate and lateralwards, to end in the paratectal BSC and other lateral targets more caudally in the alar midbrain (MRt; Cnf; Figure 15). There are nevertheless also deep terminals of the same collateral tract which reach thalamic, pretectal, and mesencephalic preoculomotor formations (IR, IC, MA3) as well as basal rubral and endorubral field loci (Ru, ERF). This last aspect was suspected by Ramón y Cajal.

Regarding the sparsity of precise information on the origins of the multiple collateral branches of the pyramidal tract commented in the Introduction, the present results confirm the separate thalamic, subthalamic, and infrasubthalamic collaterals already known to Ramón y Cajal (Figure 15) and add previously undescribed transnigral and prepontine collaterals (see Results). The transnigral fibers penetrate the SNR/SNC complex throughout its mesodiencephalic length (p3-p1 diencephalic prosomeres and m1-m2 midbrain prosomeres; see review in Puelles, 2018, 2024). It is unclear whether these transnigral fibers incorporate into the outer ifsth-py-coll component or have separate courses and targets. Maybe retrograde connectivity studies can resolve this point. On the other hand, the prepontine isthmocerebellar region, which classically was ascribed to the midbrain (Puelles, 2019), turns out to receive motor cortex pyramidal input via prepontine (hindbrain) collaterals apparently arising at rhombomere 2 level, just before the pyramidal tract enters the pons. This corroborates under this particular aspect the tagmatic separation of the midbrain (as a caudal part of the forebrain served by hypothalamic and mesodiencephalic transnigral tegmental collaterals; Puelles, 2018, 2024) from the hindbrain’s innervation by more caudal pyramidal collaterals (prepontine, etc.). Note the oculomotor nuclei are not targeted directly by cortical efferents, which rather end on preoculomotor formations.

### A paratectal (BSC) target of Ramón y Cajal’s hypothalamic collaterals

Rodent brain atlases uniformly interpret the paratectal BSC midbrain entity highlighted in this report (bed nucleus of the superior collicular brachium; Figure 15; Puelles E. et al., 2012) as a mere lateral extension of the intermediate and deep gray layers of the superior colliculus (actually, the BSC appears as a slight pial bulge whose inner structure is not clearly layered, see our Figures 13B–E, or Plates 3.6–4.1 of the Slotnick and Leonard, 1975 mouse forebrain atlas). In our analysis of mouse midbrain structures (Puelles E. et al. 2012), we commented on the fact that this paratectal locus lacks the expected collicular superficial strata and also seems devoid of optic tract projections, being covered instead by the brachium of the SC. These aspects strongly suggest this locus is not part of the superior colliculus proper.

In the past, I studied the histogenesis of the chicken and lizard homologs of the superior colliculus, the optic tectum (Puelles and Bendala, 1978; Baez et al., 2003). We observed that all tectal layers systematically develop jointly in a species-specific sequence. It is typical of mammals, in contrast to sauropsids, to develop a superficial tectal stratum via radial migrations occurring at the end of the histogenetic sequence. Its neuronal elements aggregate subpially, superficial to the optic tract fibers. This uniquely mammalian migration leaves the mammalian tectal optic stratum intercalated radially between the superficial and intermediate strata; at earlier stages, a normal marginal optic tract covering all the deeper strata is seen in mammals, exactly as in other vertebrates. There is no literature support for the idea that a lateral part of the mammalian superior colliculus lacks a superficial stratum, and if this anomaly existed, one would still expect this intermediate stratum to be covered by the early-formed marginal optic tract. No detailed histogenetic study of the mammalian superior colliculus that is equivalent to our chick and lizard analyses exists yet, but *a priori* there is no reason to assume that the production of fundamental tectal cell types, their migratory properties, and the fundamental set of strata is drastically different in mammals, irrespective of the described distinctive specialization of the emergent mammalian superficial stratum.

The suggested conventional error postulating an abnormal ‘lateral part’ of the superior colliculus devoid of retinal input (idea most recently represented by the otherwise excellent study of Benavidez et al., 2021) apparently occurred due to the superficial histologic similarity between this paratectal BSC locus and the adjacent collicular strata in Nissl-stained material. This was completed by disregarding that this locus is covered by the brachium of the superior colliculus instead of by the optic tract.

In contrast, we (Puelles E. et al., 2012) held that this paratectal locus must be a separate *paratectal griseum*. This entity would be distinct from the superior colliculus proper, even if it shares some structural, typological, and staining properties with the neighboring deep/intermediate tectal strata. Figures 13B–E show some genoarchitectonic data illustrating molecular differences between the SC and the BSC. The data of Benavidez et al., 2021 on their ‘lateral SC’ jointly with present data show that this locus is hodologically (and probably functionally) distinct from the superior colliculus. Notably, the BSC does not receive any input from visual sensory areas, nor from the frontal eye field (Zingg et al., 2017; Benavidez et al., 2021) and contains a motor representation of the whole body (indicating it strictly is not a visuomotor formation). Benavidez et al. (2021) state that “The SC.l [BSC] sits in a unique interface to integrate somatic sensorimotor information from brainstem sensory nuclei (i.e., SPVO and SPVI), cortical somatic sensorimotor areas, and cerebellum. In turn, the SC.l [BSC] sends this information to the cortex through the tecto-thalamo-cortical pathway on the one hand and projects to the motor nuclei in the lower brainstem (presumably in regulating behavior).”

These authors investigated extensively the connections of the mouse SC, combining state-of-the-art circuit mapping and viral tracing methods with computational neuroanatomical tools. Their rich results on the distribution of widely sampled cortical-tectal projections show that the SC (as defined by them, including the BSC as a lateral part) can be subdivided into four radially distinct zones along the medial-lateral axis (medial, centro-medial, centro-lateral, and lateral zones, abbreviated SC.m, SC.cm, SC.cl, and SC.l). Interestingly, each zone is defined by a unique subnetwork of cortical inputs and displays a distinct brain-wide input–output organization with significant functional implications. It turns out that the lateral SC zone (SC.l), which selectively receives *somatosensory and motor whole-body cortical input*, corresponds precisely to our BSC nucleus and is thus not according to us a part of the superior colliculus but a separate griseum (Puelles E. et al., 2012; Puelles, 2022; present data).

Note the BSC apparently is not even a stratified structure, as is the SC, since it is not obvious that what passes as its deep stratum belongs to it (see Figures 13C,D). I thus ascribe Benavidez et al. (2021) SC.l data to the BSC, just adding that the differential cortical afferents of the BSC reach it by a singular and exclusive pathway, Ramón y Cajal’s ifsth-py-coll tract. [Benavidez et al. (2021) did not comment that the cortical afferents to their SC.m, SC.cm, and SC.cl zones reach them via a suprasubthalamic -alar- transthalamic route originated in cortical areas different from the motor cortex]. The implied more precise concept of a *tectal-paratectal complex* does not alter the high factual and functional interest of the Benavidez et al. (2021) report but may qualify subtly some of their conclusions. The general somatotopic map of these somatosensory and motor projections upon the BSC shown in Benavidez et al. (2021). Their Supplementary Figure 3b reveals that cortical input from what can be interpreted as distal body parts (i.e., orofacial and distal limb parts) is what mainly reaches most of the BSC, whereas analogous truncal information apparently corresponds to the caudomedial BSC (see these authors’ Figures 1A, E, 2, 3, 5). The BSC thus seems related to motor functions of the whole body and does not specialize in oculomotor reflex control or gaze control in general, as far as the present information suggests.

### Oculomotor targets of the ifsth-py-coll tract

The deep medial component of Ramón y Cajal’s collateral tract first projects on the rostral and caudal deep parts of the prethalamic ZI next to Forel’s field (where Ramón y Cajal said his tract arborized; note Benavidez et al., 2021 also related hodologically this deep ZI part to the SC.l/BSC formation). Thereafter, these fibers continue into the thalamic tegmentum rostral to or surrounding the retroflex tract, where the *rostral interstitial nucleus of the MLF* (RI; Figure 15; a preoculomotor nucleus) is described (review in Büttner-Ennever, 2006). Caudally to the retroflex tract, the pretectal tegmentum contains spread between the MLF and nucleus ruber an analogous reticular cell group with differential physiological properties called *interstitial nucleus (of the MLF) of Ramón y Cajal*, which also receives pyramidal terminals via the medial component; this population apparently also extends into the rostral midbrain (IC; Figure 15; Ramón y Cajal first described it using reduced silver). Slightly more caudally in the midbrain, recent studies have described ‘supraoculomotor area preoculomotor cells’ (SOA in Büttner-Ennever, 2006) which apparently coincide topographically with what Paxinos and Franklin (2013) identified as ‘medial accessory oculomotor area’ (MA3; Figure 15); this area also receives dense terminals in the present material. None of the oculomotor neurons receive cortical motor input. This reveals that the medial component of the ifsth-py-coll tract forms part of what would be an indirect ‘corticonuclear’ motor connection.

### Other targets of the ifsth-py-coll tract

Along its diencephalic course, the outer component of the ifsth-py-coll tract traverses densely the PIL and REth neighborhoods of the thalamus (Figure 15). The functional meaning of any potential synaptic connections en passant on these neurons is unclear. The outer component next traverses the pretectal reticular intermediate stratum (deep to the superficial retinorecipient nuclei). Ferran et al. (2008) and Puelles E. et al. (2012) subdivided the pretectum globally into three anteroposterior microzonal subdomains distinguishable structurally and molecularly (commissural, juxtacommissural, and precommissural parts; CoP, JcP, and PcP). Dense separate patches of terminals were observed at least in our second experiment at the level of the reticular populations of JcP and CoP (JcRt and CoRt; Figure 15).

In the midbrain, apart of the BSC, the outer component fibers ramify variously in the rubral field (Ru) as well as in a dense *endorubral reticular field* (ERF) lying deep to Ru, via the bridge formed between the deep and outer components (Figure 15). The contralateral ERF also receives distinctly fibers crossing the midline (midbrain floor), suggesting it represents an important terminal field. Nothing is known yet about the physiology of this locus. Other fibers of the outer component continue caudalwards, ramifying particularly into a part of the lateral *midbrain reticular formation* (MRt) found deep to the inferior colliculus (Figure 15). Some of these caudal elements extend across the caudal end of m1 into the small preisthmic m2 mesomere, where they ramify again within its intermediate alar stratum, lying topographically under the IC (but topologically caudal to it; see Puelles and Hidalgo-Sánchez, 2023), which represents the conventional *cuneiform nucleus* (CnF; Figure 15). The latter is conceived by physiologists as the *midbrain locomotor center* (MLC). Some data indicate that this center also connects with the oculomotor nuclei (Büttner-Ennever, 2006).

All along the mesodiencephalic extent of the substantia nigra reticulata (SNR), the peduncle releases transnigral collaterals that apparently terminate partly within the SNR but finally cross likewise the SNC and seem to reach more deeply the outer component tract. It is unclear whether these transnigral elements contribute to the targets of the ifsth-py-coll tract or have separate targets.

## Data Availability

The original contributions presented in the study are included in the article/supplementary material, further inquiries can be directed to the corresponding author.

## Footnotes

^1^connectivity.brain-map.org

^2^developingmouse.brain-map.org
